# Epidemiological analysis of chronic kidney disease from 1990 to 2019 and predictions to 2030 by Bayesian age-period-cohort analysis

**DOI:** 10.1080/0886022X.2024.2403645

**Published:** 2024-09-19

**Authors:** Boqing Dong, Yuting Zhao, Jiale Wang, Cuinan Lu, Zuhan Chen, Ruiyang Ma, Huanjing Bi, Jingwen Wang, Ying Wang, Xiaoming Ding, Yang Li

**Affiliations:** a Department of Renal Transplantation, The First Affiliated Hospital of Xi’an Jiaotong University, Xi’an, China; b Department of Gynecologic Oncology, National Cancer Center/National Clinical Research Center for Cancer/Cancer Hospital, Chinese Academy of Medical Sciences and Peking Union Medical College, Beijing, China; c Chinese Academy of Medical Sciences & Peking Union Medical College, Beijing, China

**Keywords:** Chronic kidney disease, epidemiology, global burden, frontier analysis, prediction

## Abstract

**Background:**

Chronic Kidney Disease (CKD) has emerged as a significant global health issue. This study aimed to reveal and predict the epidemiological characteristics of CKD.

**Methods:**

Data from the Global Burden of Disease Study spanning the years 1990 to 2019 were employed to analyze the incidence, prevalence, death, and disability-adjusted life year (DALY) of CKD. Joinpoint analysis assessed epidemiological trends of CKD from 1990 to 2019. An age-period-cohort model evaluated risk variations. Risk factor analysis uncovered their influences on DALYs and deaths of CKD. Decomposition analysis explored the drivers to CKD. Frontier analysis evaluated the correlations between CKD burden and the sociodemographic index (SDI). A Bayesian Age-Period-Cohort model was employed to predict future incidence and death of CKD.

**Results:**

In 2019, there were 18,986,903 incident cases, 697,294,307 prevalent cases, 1,427,232 deaths, and 41,538,592 DALYs of CKD globally. Joinpoint analysis showed increasing age-standardized rates of CKD incidence, prevalence, mortality, and DALY from 1990 to 2019. High systolic blood pressure significantly contributed to CKD-related deaths and DALYs, particularly in the high SDI region. Decomposition analysis identified population growth as the primary driver of CKD incident cases and DALYs globally. Countries like Nicaragua showed the highest effective differences, indicating room for improvement in CKD management. By 2030, while incident cases of CKD were predicted to rise, the global deaths might decrease.

**Conclusions:**

The study revealed a concerning upward trend in the global burden of CKD, emphasizing the need for targeted management strategies across different causes, regions, age groups, and genders.

## Introduction

Chronic kidney disease (CKD) is a leading cause of global mortality among non-communicable diseases, characterized by a sustained decline in glomerular function and elevated levels of albuminuria [1,2]. According to the international guidelines, CKD is defined by the presence of markers of kidney damage or a glomerular filtration rate (GFR) of less than 60 mL/min per 1.73 m^2^ for three months or longer [3]. Once diagnosed with CKD, patients typically require lifelong renal replacement therapy (RRT), such as kidney transplantation or dialysis, imposing a significant burden on healthcare systems [4]. The decline in kidney function escalates the risk for cardiovascular morbidity and mortality, making CKD not only a direct cause of death but also a potent risk multiplier for other non-communicable diseases [5,6].

The epidemiology of CKD is complex and varies by region, presenting challenges for medical interventions across diverse geographical regions [7]. Variability contributing to CKD epidemiology encompasses regional disparities, differing disease definitions, and variations in the utilization of one-time testing for kidney function or albuminuria in determining CKD prevalence in epidemiological research [7]. The Global Burden of Disease Study (GBD) provides a comprehensive framework for understanding these discrepancies and predicting future trends of CKD [8]. This study aimed to offer an overview of CKD epidemiology by analyzing its incidence, prevalence, death, and disability-adjusted life year (DALY). Our goal was to offer an understanding of the changing epidemiological characteristics of CKD, facilitating better-targeted interventions and resource allocation for managing this global health challenge.

## Materials and methods

### Data sources

The data utilized in this study were obtained from the GBD 2019, which provided epidemiological data across 204 countries and territories worldwide [8]. The definition of CKD used in the GBD study differs from that in the KDIGO guidelines [1,3]. The GBD definition relies on a single measurement of estimated glomerular filtration rate (eGFR) and albumin-to-creatinine ratio (ACR), which does not meet the KDIGO criteria requiring abnormalities to persist for over three months and consider other markers of kidney damage. The incidence, prevalence, death, DALY, and risk factors of CKD were extracted using the GBD Results Tool [9–11]. The risk factors of DALYs and deaths were displayed as attributable percent. Furthermore, the computation of DALYs involved summing two components: years lived with disability (YLD) and years of life lost (YLL) [8]. Age-standardized rates (ASRs) for CKD, including age-standardized incidence rate (ASIR), age-standardized prevalence rate (ASPR), age-standardized mortality rate (ASMR), and age-standardized DALY rate (ASDR), were calculated through the direct method, with the GBD 2019 world population as the standard reference [12]. The certainty of estimates was represented by the uncertainty interval (UI). Each estimate in the GBD was calculated 1000 times. The 95% UI was then determined by taking the 25th and 975th values after sorting these 1000 results from smallest to largest. To explore differences in the burden of CKD based on per capita income, educational attainment, and fertility rates, all countries were categorized into five groups according to the sociodemographic index (SDI), including high SDI, high-middle SDI, middle SDI, low-middle SDI, and low SDI [13].

### Joinpoint analysis

We utilized Joinpoint analysis to identify significant temporal turning points in global ASR from 1990 to 2019. The average annual percent change (AAPC) was employed to describe the average annual percentage change in ASR over specified time intervals, while the annual percent change (APC) quantified the percentage variation in ASR within specific time segments.

### Age-period-cohort analysis

To illustrate the evolving trends in CKD incidence and mortality from 1990 to 2019, we conducted an age-period-cohort analysis using a tool developed by Rosenberg and his colleagues [14]. Age effects denote variations associated with individual aging processes, while period effects encompass external factors that uniformly influence outcomes across all age groups during specific calendar periods. Cohort effects represent the cumulative impact of all unique exposures experienced by a cohort from birth.

### Decomposition analysis

Decomposition analysis in epidemiology decomposed changes in health indicators, revealing the influence of factors such as population growth, aging, risk factors, or medical advancements [15]. We evaluated the contributions of various factors, including population growth, aging, and epidemiological changes. Furthermore, in analyzing DALYs, we deconstructed the epidemiological changes, by categorizing them into specific etiological factors such as glomerulonephritis, hypertension, type 1 diabetes mellitus (T1DM), type 2 diabetes mellitus (T2DM), and other causes.

### Frontier analysis

Frontier analysis was performed to identify the minimum achievable DALYs based on development levels [16]. This analysis defines a frontier that represents the lowest possible DALYs for each country or territory given its SDI. The distance from this frontier, referred to as the effective difference, indicates potential opportunities for reducing CKD DALYs in relation to a country or region’s developmental status. We employed data envelopment analysis with the free disposal hull method to delineate a non-linear frontier for ASDR using data from 1990 to 2019 [13,17,18]. To account for uncertainty, we generated 100 bootstrapped samples by randomly sampling with replacement across all countries and years. The mean CKD DALYs at each SDI value from these bootstrapped samples were then smoothed using LOESS regression [13]. Super-efficient countries were excluded to prevent outliers from distorting the frontier. Finally, we calculated the effective difference for each country or territory using 2019 SDI and ASDR data, with those below the frontier assigned a zero distance.

### The Bayesian Age-Period-Cohort model

The Bayesian Age-Period-Cohort (BAPC) model integrates age, period, and cohort effects to analyze and predict disease trends, accounting for demographic changes [19]. This model offers a nuanced approach to projecting disease trends by considering the complex interplay of demographic shifts over time. In this study, we applied the BAPC model to forecast incidence and death from 2020 to 2030. All statistical analyses and visualizations were performed using R statistical software program (version 4.1.3), with statistical significance defined as a two-sided *p* < 0.05.

## Results

### The global incidence, prevalence, death, and DALY of CKD from 1990 to 2019

**Figure 1.**
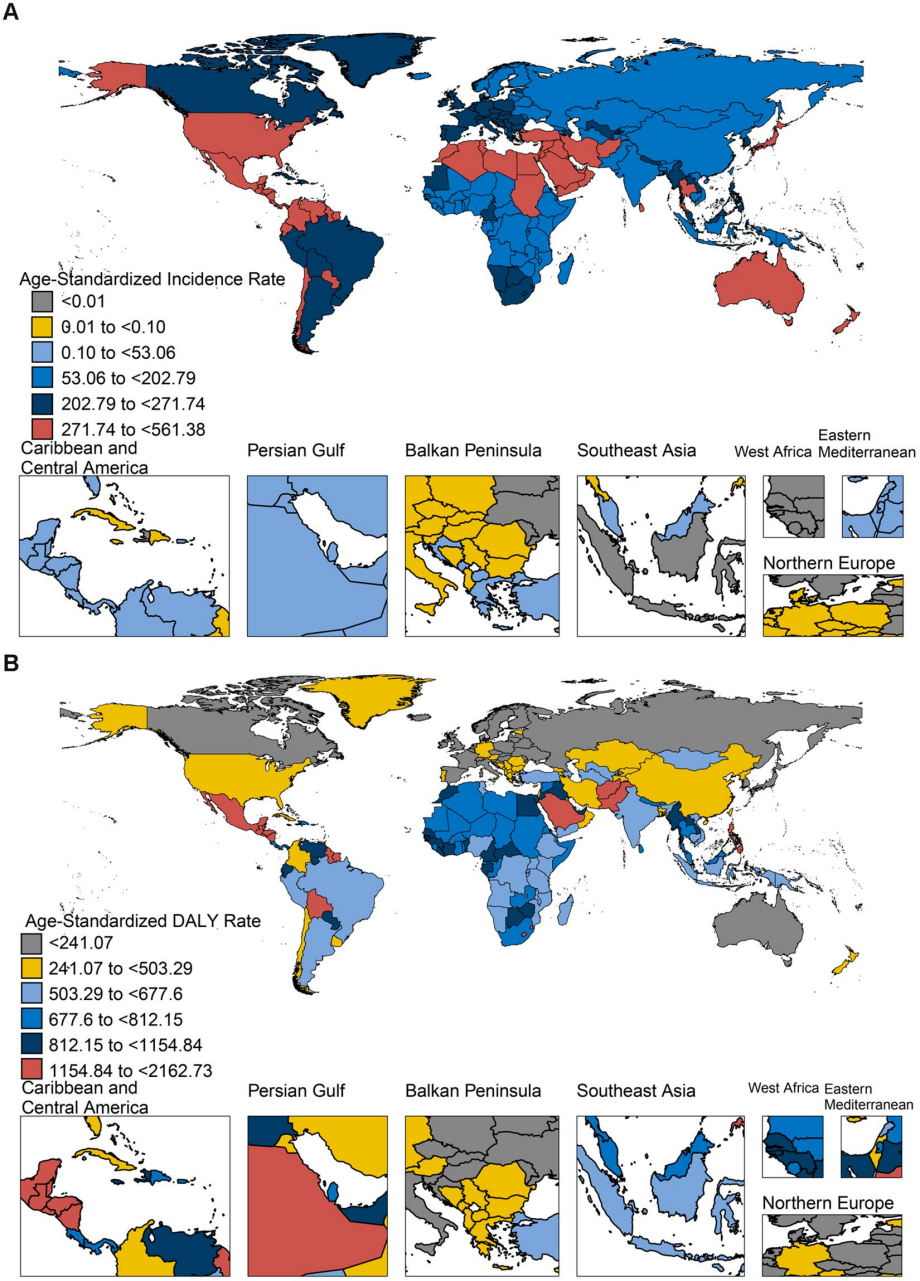
Global ASIR (A) and ASDR (B) of CKD in 2019 (per 100,000 population). ASIR: age-standardized incidence rate; ASDR: age-standardized disability-adjusted life year rate; CKD: chronic kidney disease.

The global maps of ASIR and ASDR of CKD in 2019 are shown in Figure 1(A,B), respectively. The higher ASIRs per 100,000 population (271.7–561.4) were mainly found in regions such as Australia, North America, and northern Africa, while the higher ASDRs per 100,000 population (1154.8–2162.7) were mainly found in regions such as North and South America, and the Middle East. There were 18,986,903 (95% UI = 17,556,534–20,518,156) incident cases, 697,294,307 (95% UI = 650,045,551–741,080,754) prevalent cases, 1,427,232 (95% UI = 1,313,735–1,524,548) deaths, and 41,538,592 (95% UI = 38,291,808–45,037,864) DALYs globally in 2019 (Table 1). ASIR, ASPR, ASMR, and ASDR of CKD per 100,000 population were 233.7 (95% UI = 165.7–311.1), 8600.3 (95% UI = 7107.1–10,278.2), 18.3 (95% UI = 16.3–19.8), and 515.0 (95% UI = 454.0–588.1) globally in 2019, respectively. The ASIR per 100,000 population (281.8, 95% UI = 202.5–374.4) in the high SDI region and ASPR per 100,000 population (9495.7, 95% UI = 7836.9–11,361.4) in the middle SDI region, as well as the ASMR per 100,000 population (25.3, 95% UI = 22.1–28.4) and ASDR per 100,000 population (682.6, 95% UI = 593.9–779.2) in the low SDI region, surpassed global levels and other SDI regions. In the low SDI region, the ASR per 100,000 population for ASIR (161.4, 95% UI = 112.7–216.8) and ASPR (7060.4, 95% UI = 5781.8–8514.9) showed the lowest levels. In comparison, the ASMR per 100,000 population (11.8, 95% UI = 10.4–12.9) in the high-middle SDI region and ASDR per 100,000 population (304.3, 95% UI = 260.2–358.8) in the high SDI region reported the lowest levels worldwide, respectively.

**Table 1.** The incidence, prevalence, death, DALY, and age-standardized rates, and their temporal trends of chronic kidney disease*.

	Global	High SDI	High-middle SDI	Middle SDI	Low-middle SDI	Low SDI
2019						
Incidence						
Number of cases 2019 × 10^5^ (95% UI)	189.9 (175.6–205.2)	51.5 (47.5–55.7)	41.3 (38.0–44.9)	58.7 (54.0–63.7)	28.1 (25.8–30.6)	10.1 (9.3–10.9)
ASIR 2019 per 100,000 population (95% UI)	233.7 (165.7–311.1)	281.8 (202.5–374.4)	210.6 (148.2–281.7)	235.6 (165.5–315.7)	197.1 (137.1–265.2)	161.4 (112.7–216.8)
Prevalence						
Number of cases 2019 × 10^5^ (95% UI)	6972.9 (6500.5–7410.8)	1219.5 (1145.3–1292.1)	1562.3 (1454.2–1666.5)	2398.8 (2226.4–2562.8)	1301.5 (1208.1–1395.0)	487.0 (450.5–525.1)
ASPR 2019 per 100,000 population (95% UI)	8600.3 (7107.1–10,278.2)	7368.8 (6187.7–8668.5)	8355.1 (6855.7–10,053.1)	9495.7 (7836.9–11,361.4)	8583.7 (7026.6–10,337.0)	7060.4 (5781.8–8514.9)
Death						
Number of deaths 2019 × 10^5^ (95% UI)	14.3 (13.1–15.2)	2.7 (2.4–2.9)	2.3 (2.1–2.4)	5.1 (4.6–5.5)	2.9 (2.7–3.2)	1.3 (1.1–1.4)
ASPR 2019 per 100,000 population (95% UI)	18.3 (16.3–19.8)	12.6 (10.9–13.7)	11.8 (10.4–12.9)	22.8 (20.3–25.0)	23.0 (20.2–25.6)	25.3 (22.1–28.4)
DALYs						
Number of DALYs 2019 × 10^5^ (95% UI)	415.4 (382.9–450.4)	53.8 (48.7–58.8)	60.5 (55.0–66.7)	154.4 (141.7–168.4)	99.1 (89.8–108.2)	47.3 (42.1–52.8)
ASDR 2019 per 100,000 population (95% UI)	515.0 (454.0–588.1)	304.3 (260.2–358.8)	320.7 (273.6–382.1)	622.1 (547.2–713.8)	664.4 (582.2–753.7)	682.6 (593.9–779.2)
1990–2019						
EAPC of ASIR (95% CI)	0.69 (0.67–0.72)	0.27 (0.20–0.34)	0.99 (0.97–1.00)	1.14 (1.03–1.25)	0.92 (0.85–1.00)	0.94 (0.82–1.07)
EAPC of ASPR (95% CI)	0.31 (0.30–0.32)	0.11 (0.06–0.16)	0.40 (0.34–0.46)	0.43 (0.40–0.47)	0.26 (0.15–0.38)	0.30 (0.24–0.35)
EAPC of ASMR (95% CI)	0.73 (0.61–0.86)	1.13 (0.82–1.45)	−0.16 (−0.67–0.36)	0.35 (0.11–0.60)	0.04 (−0.35–0.43)	−0.24 (−0.32–−0.17)
EAPC of ASDR (95% CI)	0.45 (0.34–0.56)	0.78 (0.52–1.05)	−0.49 (−0.86–−0.13)	0.20 (−0.08–0.48)	0.03 (−0.30–0.35)	−0.23 (−0.28–−0.17)

* Age-standardized rates were standardized with GBD 2019 world population age standard.

SDI: social-demographic index; UI: uncertainty interval; ASIR: age-standardized incidence rate; ASPR: age- standardized prevalence rate; ASMR: age- standardized mortality rate; DALYs: disability-adjusted life years; ASDR: age-standardized disability-adjusted life year rate; EAPC: estimated annual percentage change; CI: confidence interval.

According to the analysis of the trends of ASR (Table 1), global ASIR (estimated annual percentage change (EAPC) =0.69, 95% confidence interval (CI) = 0.67–0.72), ASPR (EAPC = 0.31, 95% CI = 0.30–0.32), ASMR (EAPC = 0.73, 95% CI = 0.61–0.86), and ASDR (EAPC = 0.45, 95% CI = 0.34–0.56) illustrated an increasing trend from 1990 to 2019. Within five SDI quantiles, the middle SDI region displayed the highest absolute EAPCs for ASIR (EAPC = 1.14, 95% CI = 1.03–1.25) and ASPR (EAPC = 0.43, 95% CI = 0.40–0.47). The high SDI region exhibited the highest absolute EAPCs for ASMR (EAPC = 1.13, 95% CI = 0.82–1.45) and ASDR (EAPC = 0.78, 95% CI = 0.52–1.05), indicating the most rapid increase in burden. To assess the trends in the ASR over time from 1990 to 2019, Joinpoint analysis was used (supplemental Table S1 and Figure S1). The AAPCs for ASIR, ASPR, ASMR, and ASDR were consistent with the findings from EAPCs. The ASIR and ASPR of females were consistently higher than males, while ASMR and ASDR of males were lower than those of females from 1990 to 2019. A significant temporal turning point was found in the APC for ASMR globally in 2014. From 1990 to 2014, the global ASMR gradually increased, but after this turning point, the ASMR began to decline (−0.39, 95% CI = −0.58 to −0.20, *p* < 0.001).

### Incidence, prevalence, death, and DALY of CKD by age and SDI from 1990 to 2019

From 1990 to 2019, there was a consistent increase in CKD incident and prevalent cases across all age groups (supplemental Figure S2A-B). In 2019, the highest number of incident cases was observed in the age group of 65 and older (10,479,450, 95% UI = 7,587,790–13,721,140), surpassing the incident cases in both the age groups of less than 40 years (1,702,782 95% UI = 1,084,820–2,450,023) and 40–64 years (6,804,674, 95% UI = 4,789,427–9,106,637). As for prevalent cases from 1990 to 2019, the age group of 40-64 consistently remained higher compared to the other two age groups in all periods, reaching 273,676,189 (95% UI = 220,227,928–335,989,767) in 2019.

From 1990 to 2019, the global deaths and DALYs of CKD exhibited consistent upward trajectories across all age groups (supplemental Figure S2C-D). The highest number of CKD-related deaths was observed in the age group of 65 years and older, while the age group of 40-64 exhibited the highest DALYs from 1990 to 2019. Notably, the 40–64 age group consistently had higher DALYs compared to both the younger than 40 years and older than 65 years age groups, reaching 16,463,816 (95% UI = 14,658,979–18,775,233) in 2019 globally.

### Age-period-cohort analyses on incidence and mortality of CKD

**Figure 2.**
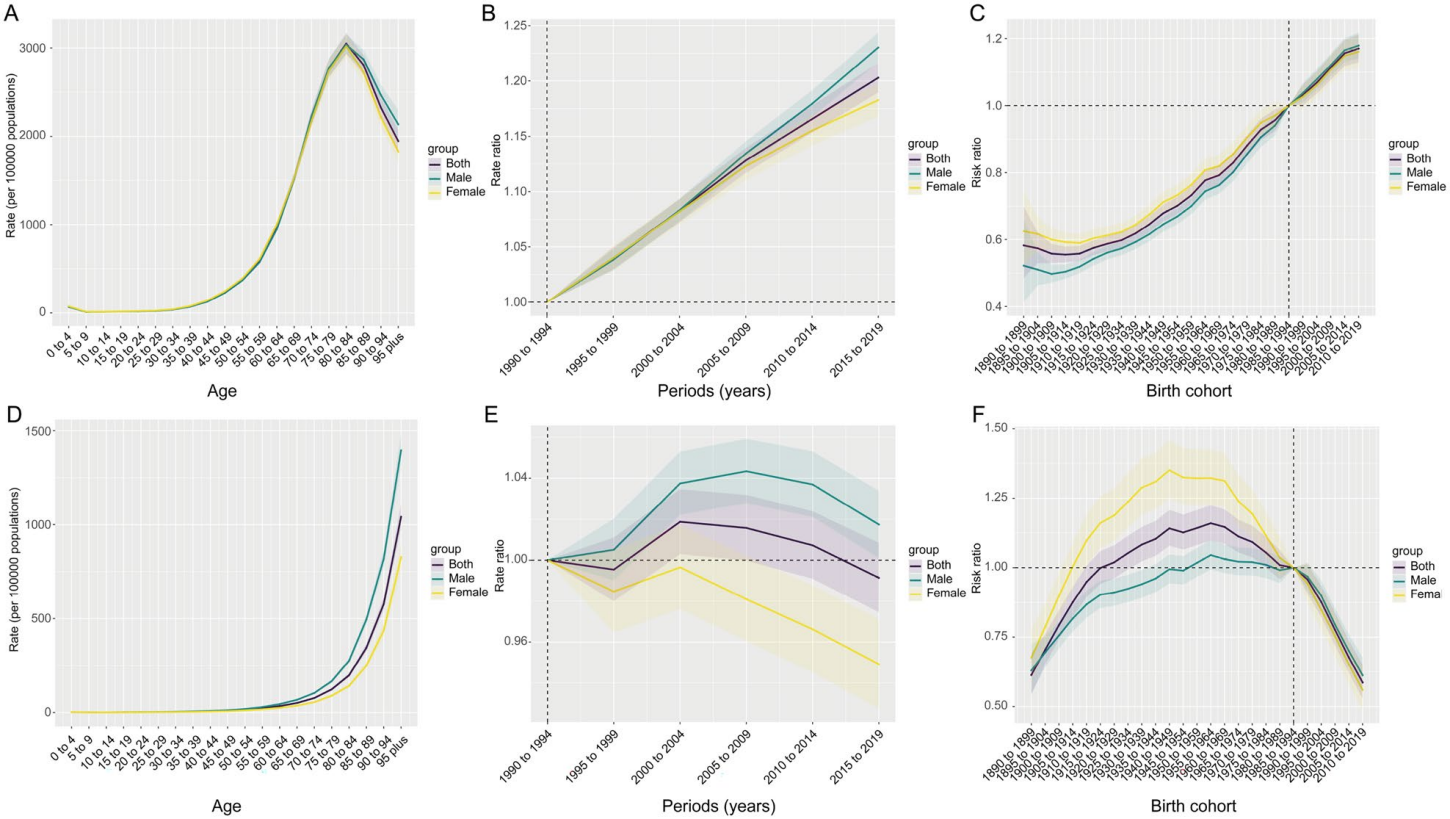
Age-period-cohort analysis of incidence (A–C) and mortality (D–F) of CKD in the globe, 1990–2019. Longitudinal age curves of incidence rate (A) and mortality rate (D). Period effects are displayed by the relative risk of incidence rate ratio (B) and mortality rate ratio (E) by comparing age-specific rates between 1990–1994 (the reference period) and 2015–2019. Cohort effects are determined by comparing the relative risk of incidence rate ratio (C) and mortality rate ratio (F) between the 1890 cohort and the 2019 cohort, with the 1985 cohort as the reference point. The dots and shaded areas represent the rate or rate ratio and their corresponding 95% CIs. CKD: chronic kidney disease; SDI: sociodemographic index; CIs: confidence intervals.

In the age-period-cohort analysis of CKD incidence among the global population from 1990 to 2019, a unimodal pattern was observed in age-specific incidence rate (Figure 2(A)). Between the ages of 0 and 84, the incidence rate of CKD gradually increased with age. However, beyond this age range, the incidence rate began to decline as individuals continued to age. In contrast to the unimodal pattern of incidence rate, the global CKD mortality rate demonstrated a consistent upward trend with increasing age (Figure 2(D)).

Regarding the period effect, the incidence rate ratio of CKD consistently increased globally from 1990 to 2019 (Figure 2(B)). In contrast, the global mortality rate ratio exhibited a complex trend, with an initial decrease followed by an increase, reaching 1.02 (95% CI = 1.00–1.03) in the years 2000 to 2004, and subsequently showing a decline (Figure 2(E)). The incidence rate ratio of CKD showed a rising trend across birth cohorts globally (Figure 2(C)). The mortality rate ratio for cohorts born after 1890 increased with each successive birth cohort until it reached 1.16 (95% CI = 1.10–1.22) for the 1955 to 1964 cohort, after which it began to steadily decline (Figure 2(F)).

### Risk factors attributable to the burden of CKD

**Figure 3.**
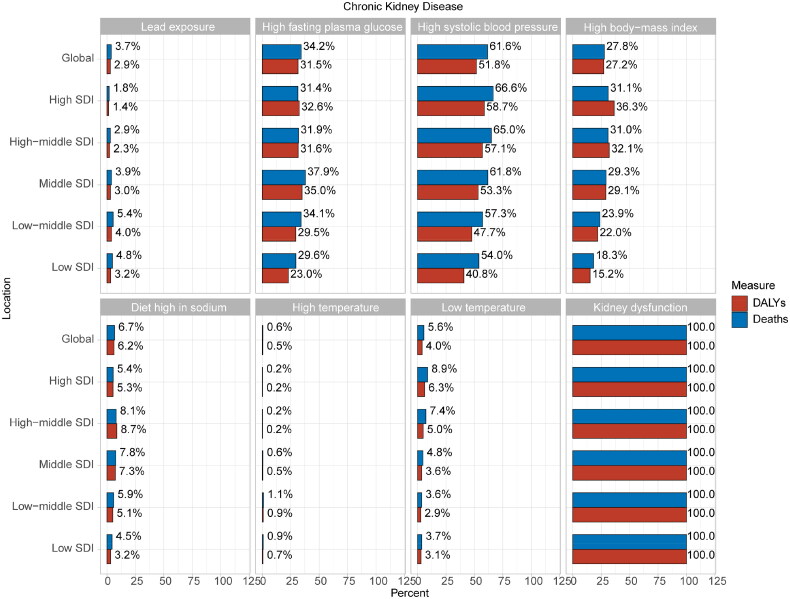
Percentage of seven risk factors for the burden of CKD. CKD: chronic kidney disease.

The contributions of risk factors to deaths and DALYs of CKD are displayed in Figure 3. Globally, high systolic blood pressure (Deaths, 61.6%; DALYs, 51.8%) was the most common contributing factor to deaths and DALYs of CKD globally, followed by high fasting plasma glucose (Deaths, 34.2%; DALYs, 31.5%) and high body-mass index (BMI) (Deaths, 27.8%; DALYs, 27.2%). In 2019, compared to other SDI regions, diet high in sodium contributed more significantly to deaths (8.1%) and DALYs (8.7%) of CKD in the high-middle SDI region, while low temperature had a greater impact on deaths (8.9%) and DALYs (6.3%) of CKD in the high SDI region.

### Decomposition analysis in epidemiology of incident cases and DALYs of CKD

**Figure 4.**
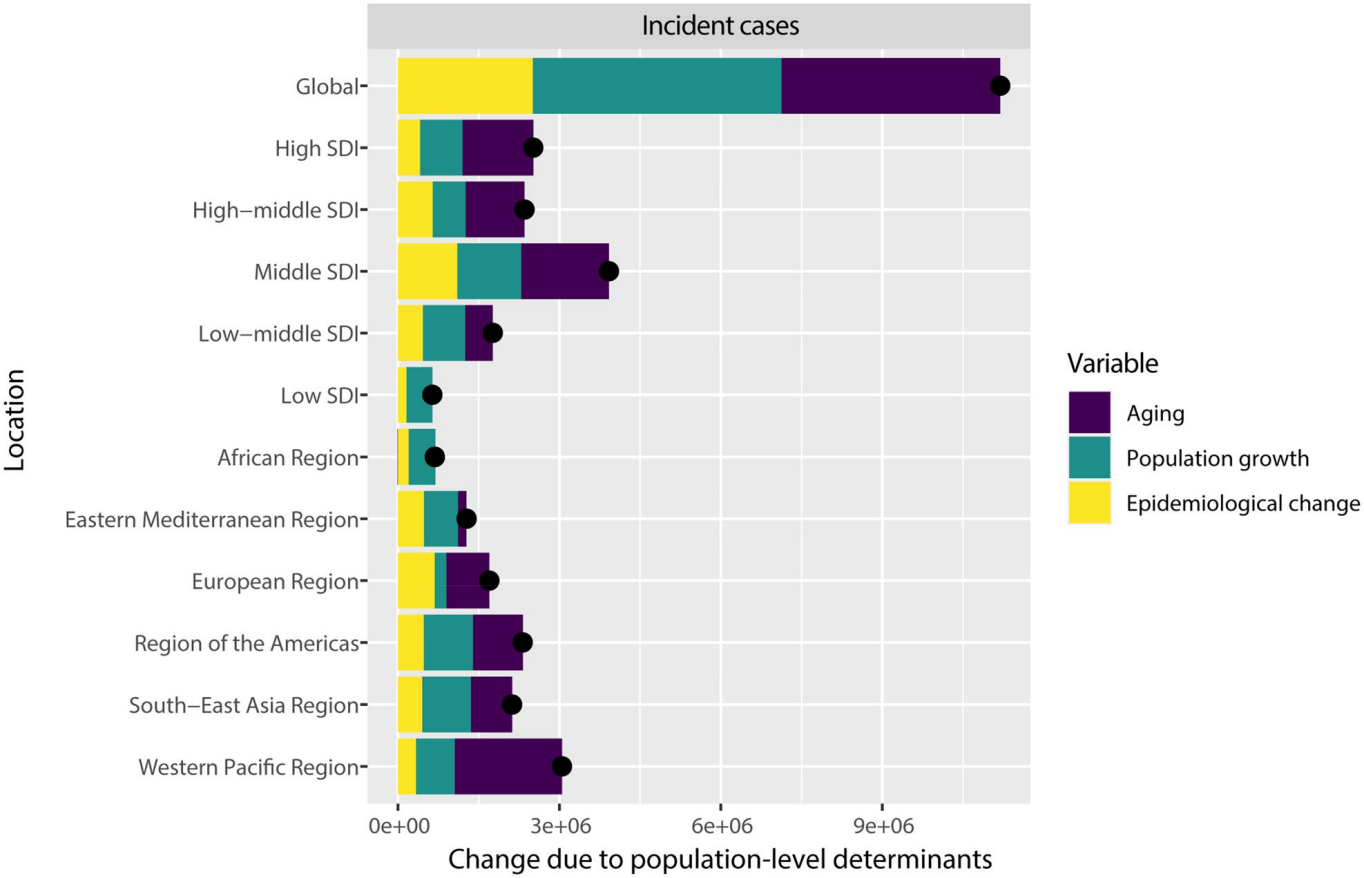
Changes in the incident cases of CKD according to the three causes from 1990 to 2019 at the global level and by SDI quintile and WHO regions. The black dot represents the overall value of incidence change contributed from all causes. CKD: chronic kidney disease; SDI: sociodemographic index; WHO: World Health Organization. Specific data is presented in supplemental Table S2.

A decomposition analysis of CKD incident cases was conducted globally, across five SDI regions, and within six WHO regions to investigate the impact of aging, population growth, and epidemiologic change from 1990 to 2019. On a global scale, population growth was the primary driver, contributing to 41.31% of the increased incident cases between 1990 and 2019 (Figure 4 and supplemental Table S2).

**Figure 5.**
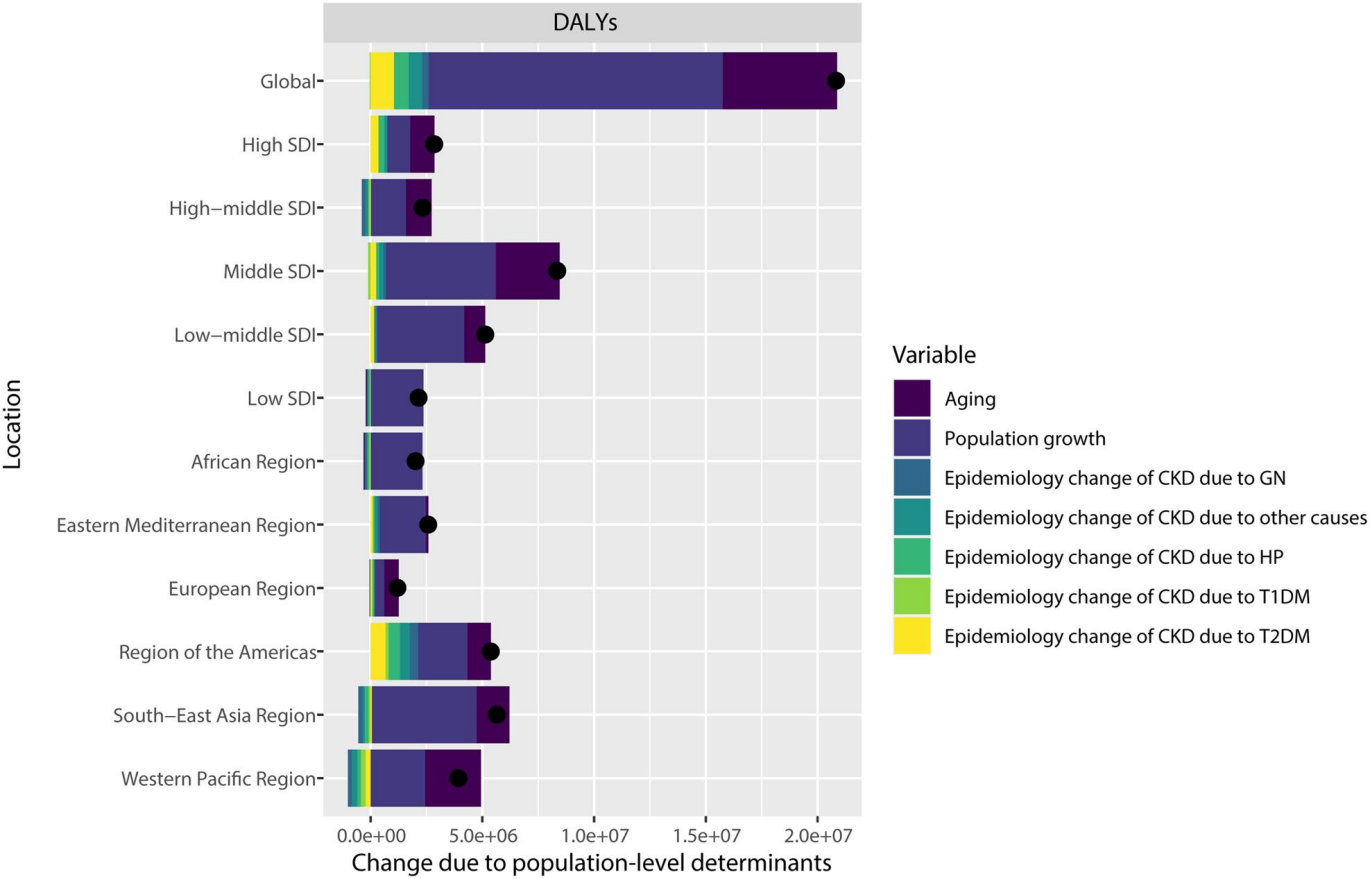
Changes in DALYs of CKD according to seven causes from 1990 to 2019 at the global level and by SDI quintile and WHO regions. The black dot represents the overall value of DALYs change contributed from all causes. DALYs: disability-adjusted life years; CKD: chronic kidney disease; SDI: sociodemographic index; WHO: World Health Organization; T1DM: type 1 diabetes mellitus; T2DM: type 2 diabetes mellitus; GN: glomerulonephritis; HP: hypertension. Specific data is presented in supplemental Table S3.

As shown in Figure 5 and supplemental Table S3, population growth was also the primary driver of the global increase in DALYs, contributing 63.20% to the rise in DALYs from 1990 to 2019. However, in the high SDI region (38.13%), Western Pacific Region (63.79%), and European Region (53.91%), aging was the predominant driver. Conversely, in the low SDI region (−2.20%) and African Region (−2.60%), aging was a negative driver for DALYs. To further analyze the driving forces of DALYs between 1990 and 2019 by different etiologies, epidemiological changes were decomposed into five detailed causes of CKD. Globally, T2DM was the most significant driver among the five diseases, accounting for 5.02%, followed closely by hypertension at 3.14%. The relative contribution of T2DM to DALYs varied across different SDI regions, being higher in high SDI (11.69%), middle SDI (2.94%), and low-middle SDI regions (2.88%). In comparison, T2DM was a negative driver in the low SDI region (−0.68%).

### Frontier analysis of ASDR in CKD across different countries and regions

**Figure 6.**
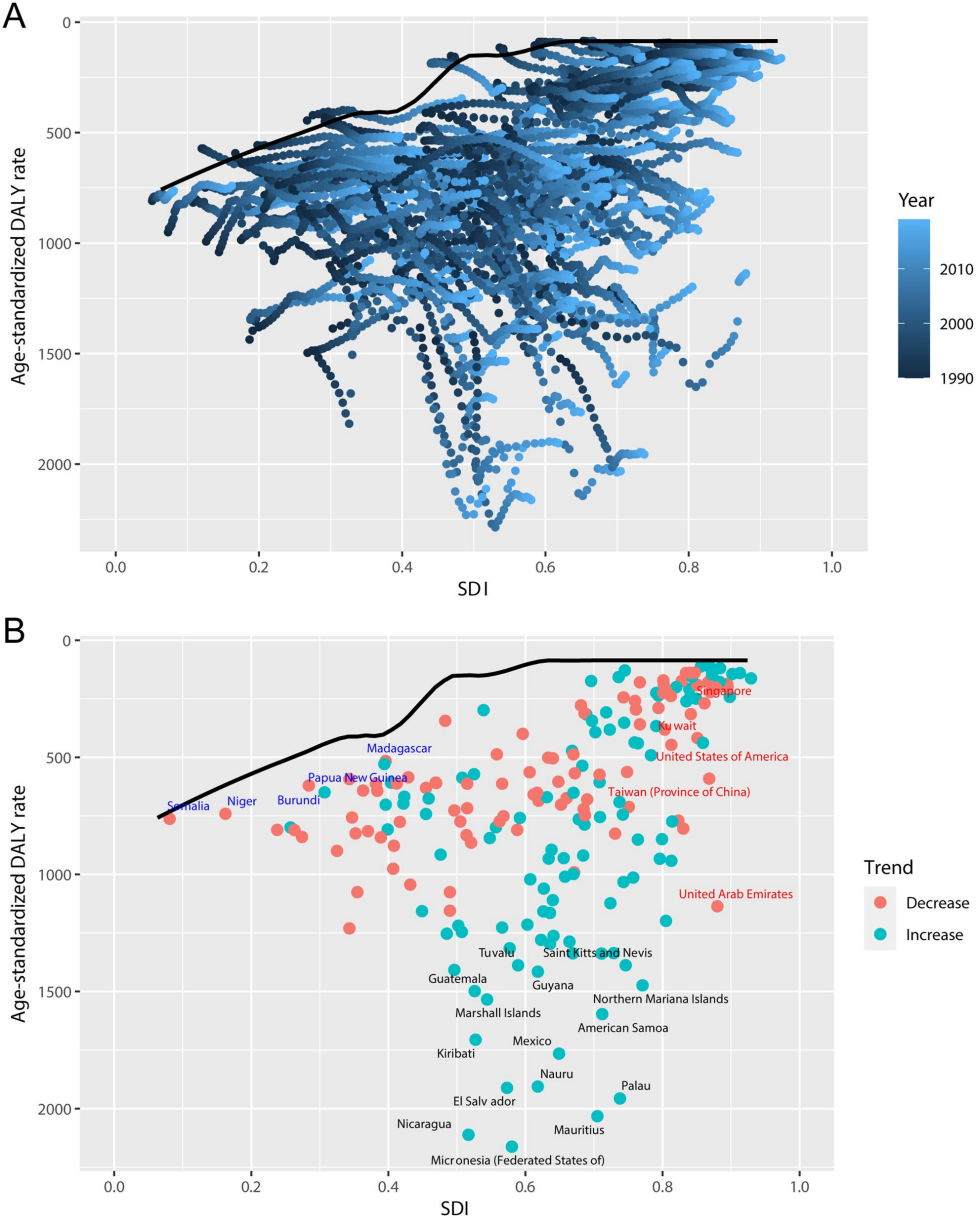
(A) Frontier analysis of CKD based on SDI and ASDR from 1990 to 2019. The color scale represents the years from 1990, depicted in black, to 2019, shown in blue. The frontier is delineated in a solid black color. (B) Frontier analysis based on SDI and CKD ASDR in 2019. The frontier line is black; countries and territories are represented as dots. The top 15 countries with the most considerable effective difference of ASDR from the frontier line are marked in black words; examples of the countries with low SDI (<0.5) and low effective difference are labeled in blue (e.g., Somalia, Niger, Burundi, Papua New Gui). Examples of countries and territories with high SDI (>0.85) and relatively high effective distance for their level of development are labeled in red (e.g., United Arab Emirates, Taiwan (Province of China), United States of America, Kuwait, Singapore). Red dots indicate an increase in ASDR of CKD from 1990 to 2019; blue dots indicate a decrease in ASDR of CKD between 1990 and 2019. CKD: chronic kidney disease; SDI: sociodemographic index; ASDR: age-standardized disability-adjusted life year rate. Specific data is presented in supplemental Table S4.

To investigate changing trends in CKD burden across various territories, a frontier analysis of ASDR spanning 204 countries and regions worldwide was conducted from 1990 to 2019. This analysis, which uses ASDR as a key metric for understanding the impact of CKD, delineates countries and regions along a frontier line based on their respective SDI levels (Figure 6(A)). The top five countries with the greatest effective difference from the frontier, ranging from 1816.76 to 2033.74 (Figure 6(B) and supplemental Table S4), included Micronesia (Federated States of), Nicaragua, Mauritius, Palau, and Nauru. These nations exhibited a disproportionately higher ASDR compared to countries with similar sociodemographic resources. Conversely, the top five countries with the lowest ASDR within their development spectrum, with effective differences ranging from 0.00 to 42.51 included Somalia, Finland, Iceland, San Marino, and Belarus.

### Prediction of incidence and deaths of CKD using the Bayesian Age-Period-Cohort model

**Figure 7.**
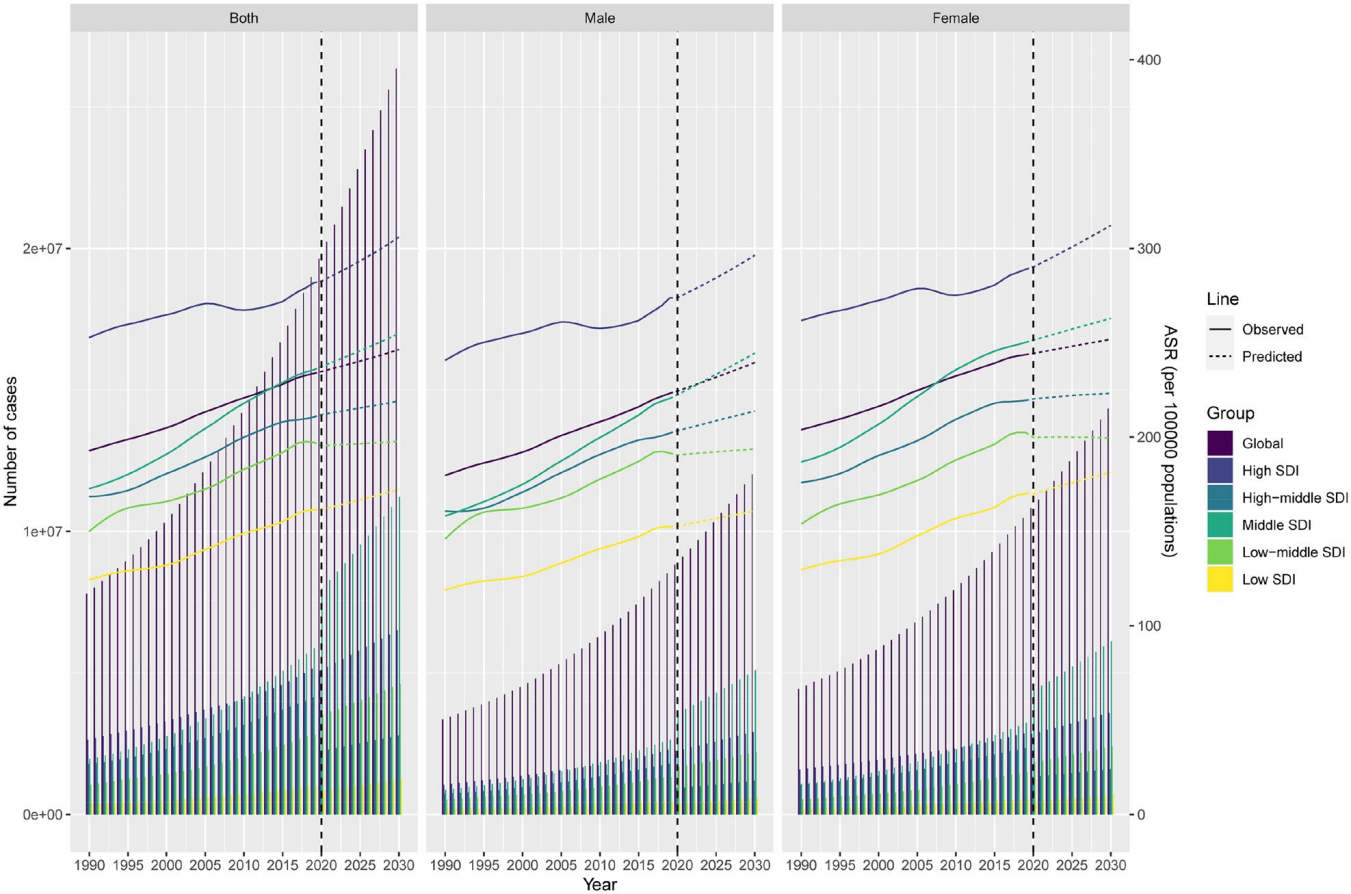
The trends of incident cases and ASIR in CKD in the globe and five SDI regions by sex, 1990–2030. ASIR: age-standardized incidence rate; CKD: chronic kidney disease; SDI: sociodemographic index; ASR: age-standardized rate. Specific data is presented in supplemental Table S5.

The BAPC model was employed to predict the evolving global trends in the incidence of CKD from 2020 to 2030 (Figure 7 and supplemental Table S5). The global incidence rate per 100,000 population is expected to increase from 234.72 (95% CI = 135.40–586.10) in 2020 to 246.36 (95% CI = 0.93–2973.94) in 2030. The global incident cases will also increase from 19,627,543 (95% CI = 19,332,649–19,922,437) in 2020 to 26,346,589 (95% CI = 22,303,728–30,389,451) in 2030. This trend is consistent across different genders and SDI. Subsequently, based on mortality rate and deaths from 1990 to 2019, predictions were made for the global death trends of CKD from 2020 to 2030 (supplemental Figure S3 and Table S6). By 2030, the global mortality rate per 100,000 population will decrease from 18.45 (95% CI = 1.23–543.11) in 2020 to 17.41 (95% CI = 0.00–5203.99) in 2030. Meanwhile, the global deaths were expected to increase from 1,483,812 (95% CI = 1,452,296–1,515,327) in 2020 to 1,924,241 (95% CI = 1,541,145–2,307,336) in 2030.

In the predicted year 2030, the global incidence rate per 100,000 population is expected to be higher in females (251.78, 95% CI = 213.35–290.21) than in males (239.44, 95% CI = 202.48–276.41), with the number of incident cases also higher in females (14,330,860, 95% CI = 12,143,305–16,518,416) compared to males (12,015,729, 95% CI = 10,160,423–13,871,035). However, the mortality rate per 100,000 population is anticipated to be higher in males (20.82, 95% CI = 16.90–24.74) than in females (15.58, 95% CI = 12.30–18.85), with the number of deaths also expected to be greater in males (991,441, 95% CI = 804,591–1,178,290) than in females (932,800, 95% CI = 736,554–1,129,046).

## Discussion

While previous studies have utilized GBD data to explore the epidemiology of CKD, many were either descriptive or focused on specific aspects of the disease (supplemental Table S7). For instance, Hu et al. concentrated on glomerulonephritis-induced CKD, emphasizing its significant burden in low SDI regions [20]. Similarly, Qing et al. and Feng et al. mapped the global burden of CKD using GBD 2019 data but did not investigate the potential drivers of CKD burden growth or predict future trends [21,22]. In contrast, our study provides more comprehensive analysis covering 204 countries and territories, offering a global perspective on CKD’s burden. We employed a variety of statistical methods, including decomposition analysis, frontier analysis, and BAPC modeling. These approaches allowed us to identify the primary drivers of CKD burdens, assess the efficiency of CKD management in relation to sociodemographic development, and predict future trends in CKD incidence and mortality. From 1990 to 2019, there was a notable rise in the burden of CKD, evidenced by its increased incidence, prevalence, mortality, and DALYs. On a global scale, in 2019, there were over 18 million new cases of CKD, reflecting a 69% increase, with 697 million prevalent cases, marking a 31% rise. Additionally, CKD accounted for nearly 1.4 million deaths, signifying a 73% surge, and resulted in 41 million years of healthy life lost, representing a 45% uptick. The BAPC model predicted a consistent increase of incidence and death from 2020 to 2030, reinforcing the persistent need for effective CKD management on a global scale.

In 2019, high systolic blood pressure was identified as a major risk factor of deaths and DALYs attributed to CKD globally. This finding is consistent with previous prospective observational studies that demonstrated a strong association between elevated blood pressure and the risk of CKD and end-stage renal disease (ESRD) [23,24]. Additionally, decomposition analysis highlighted hypertension as a key contributor to the global increase in CKD-related DALYs. These findings emphasize the critical importance of blood pressure management in the prevention and treatment of CKD. Hypertension and CKD often coexist, with poor blood pressure control significantly elevating the risk of developing cardiovascular and cerebrovascular complications [25,26]. The KDIGO guidelines recommend intensified antihypertensive therapy for CKD patients, targeting blood pressure control at 120/80 mmHg [27], while the European Renal Association advocated the use of ambulatory blood pressure monitoring with a target of 130/80 mmHg in patients on chronic dialysis [28]. Although intensified blood pressure control has not been conclusively shown to improve renal function, it reduces the incidence of cardiovascular complications [26,29]. Public health policies should prioritize early detection and management of hypertension among CKD patients, incorporating regular blood pressure monitoring and individualized antihypertensive therapy. Furthermore, public health initiatives should encourage lifestyle interventions, such as sodium reduction and fluid management. Given the challenges with patient compliance, especially concerning sodium intake, educating CKD patients to adopt and maintain these lifestyle changes is necessary [28]. Effective management of hypertension through a combination of pharmacological and non-pharmacological strategies can significantly reduce the burden of CKD and improve patient outcomes.

High fasting plasma glucose emerged as another significant risk factor contributing to the global burden of CKD in 2019. Decomposition analysis further identified T2DM as the primary driver of the increasing burden from 1990 to 2019. According to the International Diabetes Federation data in 2021, the global adult population with diabetes has reached 537 million and is expected to continue rising [30]. In the United States, nearly one-fourth of healthcare costs were related to T2DM, with a significant portion of these expenses attributable to T2DM-related CKD [31]. These results indicated that the burden of T2DM and T2DM-related CKD has been increasing year by year, leading to significant personal and social burdens. Public health policies for T2DM patients should emphasize early detection and glycemic control to prevent the development of CKD. Regular kidney function screening and provision of renal protective treatments, such as ACE inhibitors and SGLT2 inhibitors, could reduce CKD incidence [32]. Diabetic nephropathy (DN) is a complication of diabetic microvascular disease and has become one of the leading causes of ESRD. To prevent the progression of DN and the subsequent cardiovascular morbidity and mortality, it is crucial to implement personalized treatment, including strict blood glucose control, blood pressure management through RAAS inhibitors, aspirin, and lipid-lowering drugs [33]. Our study also found that in the low SDI region, T2DM was a negative driver of the increase in CKD burden from 1990 to 2019. It is necessary to cautiously interpret this result, as data collection and T2DM diagnosis in the low SDI region may be inadequate. Additionally, due to a lack of medical resources, T2DM patients may die prematurely from other infectious diseases before progressing to CKD, masking T2DM’s contribution to CKD burden [34]. Therefore, when formulating CKD-related health policies in the low SDI region, it is important to consider data quality and the most urgent health challenges faced by the local population.

From 1990 to 2019, ASIR and ASPR of CKD continued to rise for both males and females, while the ASDR and ASMR have shown a declining trend. Notably, the ASIR has consistently been higher in females. In contrast, males had higher ASDR and ASMR, indicating their worse outcomes. These gender differences were also found in the results by the BAPC model predictions, which indicated a higher incidence rate and incident cases of CKD in females, while mortality rates and deaths were higher in males. Previous epidemiological studies have also shown that CKD prevalence was generally higher in females, but kidney function declined faster in males [35,36]. The underlying reasons for this epidemiological difference between genders in CKD remain largely unexplored. Potential factors include physiological and lifestyle differences, such as longer life expectancy in females, the impact of pregnancy and estrogen, the detrimental effect of testosterone on male renal function, and unhealthy lifestyle choices among males [37]. It is noteworthy that many studies have revealed that in some countries, there are more male CKD patients undergoing RRT than females [38]. These data suggested, on the one hand, that CKD progresses more rapidly in males, and on the other hand, it indicated that females may tend to opt for conservative treatment modalities. Data from deceased kidney transplantation in the United States indicated that both the absolute number and transplantation rate of females were lower than those of males [39]. The difference in RRT between genders needs to be interpreted cautiously, and it cannot be directly concluded that there was unfair CKD management between males and females because most of this data originated from the United States and lacked high-quality research reports from other regions. Given the epidemiological differences in CKD between genders, gender-specific preventive, diagnosis, and management measures for CKD are crucial. For females, early detection and prevention are crucial, with an emphasis on educating them about CKD risks and encouraging regular screenings to reduce higher incidence rates. In contrast, male-focused strategies should prioritize managing complications, particularly cardiovascular issues, and promoting lifestyle interventions to address the faster disease progression and higher mortality rates. Additionally, policies should ensure equitable access to RRT and consider gender disparities in kidney transplantation.

In 2019, the highest number of incidence cases was seen in individuals aged 65 and older, while this age group consistently displayed the highest CKD-related mortality. The Age-Period-Cohort analysis of CKD showed that the mortality rate of CKD consistently rose with age. Decomposition analysis identified aging as the primary driver of the increasing burden in the high SDI region. According to the results from the US Renal Data System, the prevalence of CKD among individuals aged 65 and older was 33.2% in 2020, compared to 9% among younger adults [40]. Additionally, with the global increase in life expectancy, it is projected that by 2050, people aged 65 and older will make up more than 16% of the global population, with about two-thirds of those over 60 years old living in low-middle income countries [41]. This poses significant challenges for the formulation of public health policies in these countries. There are several potential reasons why older adults are more susceptible to CKD, including the loss of functional nephrons and the common presence of chronic conditions such as cardiovascular diseases and atherosclerosis [42]. Early vascular aging, characterized by accelerated arterial stiffness and endothelial dysfunction, plays a critical role in CKD pathogenesis among the elderly [43]. Early vascular aging not only contributes to the progression of CKD but also exacerbates cardiovascular complications, further increasing mortality risk in this population. To enhance CKD management in the elderly, public health policies should consider adopting age-specific eGFR thresholds and biomarkers to improve diagnosis and stratification. Incorporating routine assessments of arterial stiffness and endothelial function into CKD screening could also improve risk stratification and prevent cardiovascular complications. Finally, comprehensive pharmacological treatments, including anti-aging drugs and integrated antihypertensive treatments for arterial stiffness, are essential to further reducing mortality and improving outcomes in the elderly population [44]. In countries where CKD medications are unaffordable, dietary and behavioral therapies like calorie restriction, regular exercise, and a protein-restricted diet can promote healthy aging and reduce CKD progression [45,46].

The epidemiological features of CKD vary globally, with the United States having a 14% prevalence, driven by factors like T2DM, hypertension, and obesity [47,48]. In East Asia, CKD also poses a significant health challenge, with an estimated prevalence of 28.7% [49]. Besides diabetes and high blood pressure, major contributors to CKD in Eastern Asia include exposure to renal toxic substances and high-salt diets [50–52]. The SDI, covering per capita income, education levels, and fertility rates, is important for understanding the epidemiological characteristics of CKD globally. Overall, as SDI increased, ASIR rose, while ASMR and ASDR decreased from 1990 to 2019. This phenomenon has complex reasons, including disparities in healthcare funding, diagnostic criteria, and data entry quality [53]. Decomposition analysis revealed differences in CKD burden increases among regions with different SDI levels. In high, high-middle, and middle SDI regions, aging is the primary driver of CKD incidence increase, whereas in low-middle and low SDI regions, population growth is the main factor. Regarding the increase in CKD-related DALYs, besides aging being the primary driver in high SDI regions, population growth predominantly drives this increase in other SDI regions, particularly in low SDI regions (109.32%). Considering these findings, attention to CKD in elderly populations is warranted in high SDI regions, along with encouraging optimized fertility rates [2]. Globally, T2DM and hypertension play the most significant role in increasing CKD-related DALYs in high SDI regions. Therefore, emphasis should be placed on managing these comorbidities and promoting healthy lifestyle habits in these regions [54]. In contrast, countries with lower SDI levels exhibit lower social development, making it challenging to meet the healthcare needs of CKD. The intervention measures should prioritize disease care, treatment management, and improvement of environmental sanitation conditions [55]. Frontier analysis assessed the burden of CKD in different countries and regions, identifying areas for improvement based on SDI evaluation. Our analysis indicated that some high SDI countries exhibit higher disease burdens, such as the United Arab Emirates, Qatar, and Guam, necessitating targeted healthcare policies to ensure appropriate CKD management. Conversely, high-SDI countries like Switzerland, Norway, and Monaco showed minimal disparities between frontier DALYs and ASDR, suggesting progress in CKD management and efficient utilization of healthcare resources. These interplays between socio-economic development, healthcare accessibility, and CKD burden underscored the demand for customized healthcare strategies in regions of varying developmental stages to address the evolving burden of CKD.

Our study has several limitations. First, the definition of CKD in GBD relies on a single measurement of eGFR and ACR, which allows for broader and more inclusive estimates of CKD prevalence, making it useful for capturing a wide range of cases globally. However, this approach may include individuals with temporary or reversible kidney issues, as it does not meet the KDIGO criteria requiring abnormalities to persist for over three months. Additionally, the GBD definition does not consider other kidney damage markers beyond ACR, which are often unreported in the epidemiological studies that inform disease prevalence estimates. Second, variations in data quality and regional registration systems within the GBD dataset may introduce biases and affect the precision of our results. While the GBD study provides a comprehensive and standardized approach to estimating risk factor exposure and attributable burdens, it is based on aggregated data, limiting the application of traditional methods for controlling confounding factors. Third, the uncertainty inherent in the BAPC model can result in wider confidence intervals. This is due to the model’s reliance on past trends, which may be increasingly affected by data sparsity and variability over longer time horizons. Additionally, unanticipated health events, such as the emergence of Corona Virus Disease 2019 and other infectious diseases, can disrupt healthcare systems and population health trends, impacting the accuracy of our predictions.

## Conclusion

In conclusion, this study examined the epidemiological trends of CKD from 1990 to 2019 and projected them through 2030. The findings underscore the escalating global burden of CKD, driven by age, SDI, lifestyle changes, and gender. The Age-Period-Cohort analysis highlights the importance of age-specific preventive strategies. Effective management of blood pressure and blood glucose is essential, particularly in high SDI regions. Public health policies should prioritize early detection and the integration of CKD prevention into broader health initiatives, while addressing regional and demographic disparities to mitigate CKD’s future impact and improve global health outcomes.

## Supplementary Material

Supplementary_Material new.doc

## Data Availability

This work used the resource from the Global Burden of Disease Study (https://www.healthdata.org/research-analysis/gbd). All resources are free to obtain online.
