# Supplementary material for: Epidemiological analysis of chronic kidney disease from 1990 to 2019 and predictions to 2030 by Bayesian age-period-cohort analysis
Source: Ren Fail. 2024 Sep 19;46(2):2403645. doi: 10.1080/0886022X.2024.2403645 (PMC11413963; doi:10.1080/0886022X.2024.2403645)
Supplement: Supplementary_Material new.doc [file IRNF_A_2403645_SM1119.doc]

**Supplementary material**

**Table S1. Joinpoint analysis of global CKD incidence, prevalence, mortality, and DALYs rates, GBD study 2019, 1990-2019.**

**Table S2. Changes in incident cases according to population-level determinants from 1990 to 2019 globally and by SDI levels as well as WHO regions.**

**Table S3. Changes in DALYs according to cause of CKD from 1990 to 2019 globally and by SDI levels as well as WHO regions.**

**Table S4. Frontier DALYs, and effective difference by country or territory.**

**Table S5. BAPC prediction of incidence of CKD by sex and SDI in 2020-2030.**

**Table S6. BAPC prediction of death of CKD by sex and SDI in 2020-2030.**

**Table S7. Previous studies using the GBD database to analyze the epidemiology of CKD.**

**Figure S1. Joinpoint analysis of (A) ASIR, (B) ASPR, (C) ASMR, and (D) ASDR of CKD in the globe from 1990 to 2019.** ASIR, age-standardized incidence rate; ASPR, age-standardized prevalence rate; ASMR, age-standardized mortality rate; ASDR, age-standardized disability-adjusted life year rate; CKD, chronic kidney disease.

**Figure S2. The incident cases (A), prevalent cases (B), deaths (C) and DALYs (D) of CKD in the globe by age, 1990-2019.** DALYs, disability-adjusted life years; CKD, chronic kidney disease.

**Figure S3. The trends of deaths and ASMR in CKD in the globe and five SDI regions by sex, 1990-2030.** ASMR, age-standardized mortality rate; CKD, chronic kidney disease; SDI, sociodemographic index; ASR, age-standardized rate.

**Table S1. Joinpoint analysis of global CKD incidence, prevalence, mortality, and DALYs rates, GBD study 2019, 1990-2019.**

| Joinpoint Trends for Age-standardized Rates* | | | | | | | | | | | | | | | | | | | | | | | | | | |
| --- | --- | --- | --- | --- | --- | --- | --- | --- | --- | --- | --- | --- | --- | --- | --- | --- | --- | --- | --- | --- | --- | --- | --- | --- | --- | --- |
|  |  |  |  | Trend 1 | | |  | Trend 2 | | |  | Trend 3 | | |  | Trend 4 | | |  | Trend 5 | | |  | Trend 6 | | |
|  | AAPC (95% CI) | *P^a^* |  | Years | APC (95% CI) | *P* |  | Years | APC (95% CI) | *P* |  | Years | APC (95% CI) | *P* |  | Years | APC (95% CI) | *P* |  | Years | APC (95% CI) | *P* |  | Years | APC (95% CI) | *P* |
| Total |  |  |  |  |  |  |  |  |  |  |  |  |  |  |  |  |  |  |  |  |  |  |  |  |  |  |
| Incidence | 0.67 (0.65 to 0.69) | < 0.001 |  | 1990-2001 | 0.62 (0.61 to 0.63) | < 0.001 |  | 2001-2004 | 0.90 (0.76 to 1.03) | < 0.001 |  | 2004-2009 | 0.70 (0.66 to 0.74) | < 0.001 |  | 2009-2014 | 0.63 (0.59 to 0.68) | < 0.001 |  | 2014-2017 | 0.75 (0.62 to 0.89) | < 0.001 |  | 2017-2019 | 0.48 (0.34 to 0.61) | < 0.001 |
| Prevalence | 0.31 (0.30 to 0.32) | < 0.001 |  | 1990-2002 | 0.34 (0.33 to 0.34) | < 0.001 |  | 2002-2011 | 0.28 (0.27 to 0.29) | < 0.001 |  | 2011-2014 | 0.53 (0.45 to 0.60) | < 0.001 |  | 2014-2017 | 0.27 (0.20 to 0.34) | < 0.001 |  | 2017-2019 | 0.03 (-0.04 to 0.10) | 0.403 |  | - | - | - |
| Mortality | 0.45 (0.40 to 0.50) | < 0.001 |  | 1990-1995 | 0.39 (0.23 to 0.55) | < 0.001 |  | 1995-2003 | 1.27 (1.19 to 1.36) | < 0.001 |  | 2003-2014 | 0.25 (0.20 to 0.31) | < 0.001 |  | 2014-2019 | -0.39 (-0.58 to -0.20) | < 0.001 |  | - | - | - |  | - | - | - |
| DALYs | 0.22 (0.14 to 0.31) | < 0.001 |  | 1990-1995 | 0.20 (0.08 to 0.32) | 0.003 |  | 1995-2001 | 0.96 (0.85 to 1.07) | < 0.001 |  | 2001-2005 | 0.46 (0.20 to 0.71) | 0.002 |  | 2005-2014 | -0.07 (-0.13 to -0.01) | 0.025 |  | 2014-2017 | 0.22 (-0.39 to 0.83) | 0.452 |  | 2017-2019 | -1.04 (-1.69 to -0.39) | 0.004 |
| Male |  |  |  |  |  |  |  |  |  |  |  |  |  |  |  |  |  |  |  |  |  |  |  |  |  |  |
| Incidence | 0.76 (0.74 to 0.77) | < 0.001 |  | 1990-1993 | 0.85 (0.80 to 0.91) | < 0.001 |  | 1993-2000 | 0.61 (0.60 to 0.63) | < 0.001 |  | 2000-2005 | 0.91 (0.88 to 0.94) | < 0.001 |  | 2005-2014 | 0.71 (0.70 to 0.72) | < 0.001 |  | 2014-2017 | 0.91 (0.81 to 1.01) | < 0.001 |  | 2017-2019 | 0.69 (0.59 to 0.79) | < 0.001 |
| Prevalence | 0.30 (0.29 to 0.30) | < 0.001 |  | 1990-1992 | 0.43 (0.35 to 0.51) | < 0.001 |  | 1992-2005 | 0.34 (0.34 to 0.35) | < 0.001 |  | 2005-2010 | 0.26 (0.24 to 0.28) | < 0.001 |  | 2010-2017 | 0.40 (0.39 to 0.41) | < 0.001 |  | 2017-2019 | 0.24 (0.16 to 0.31) | < 0.001 |  | - | - | - |
| Mortality | 0.35 (0.30 to 0.40) | < 0.001 |  | 1990-1995 | 0.30 (0.10 to 0.50) | 0.005 |  | 1995-2003 | 1.20 (1.10 to 1.30) | < 0.001 |  | 2003-2014 | 0.19 (0.14 to 0.24) | < 0.001 |  | 2014-2019 | -0.59 (-0.78 to -0.41) | < 0.001 |  | - | - | - |  | - | - | - |
| DALYs | 0.17 (0.08 to 0.26) | < 0.001 |  | 1990-1995 | 0.18 (0.02 to 0.34) | 0.033 |  | 1995-2003 | 0.97 (0.88 to 1.06) | < 0.001 |  | 2003-2008 | 0.23 (0.03 to 0.43) | 0.030 |  | 2008-2017 | -0.10 (-0.18 to -0.02) | 0.014 |  | 2017-2019 | -0.95 (-1.79 to -0.11) | 0.029 |  | - | - | - |
| Female |  |  |  |  |  |  |  |  |  |  |  |  |  |  |  |  |  |  |  |  |  |  |  |  |  |  |
| Incidence | 0.62 (0.61 to 0.63) | < 0.001 |  | 1990-1996 | 0.57 (0.55 to 0.58) | < 0.001 |  | 1996-2001 | 0.65 (0.63 to 0.68) | < 0.001 |  | 2001-2004 | 0.82 (0.75 to 0.90) | < 0.001 |  | 2004-2009 | 0.69 (0.67 to 0.72) | < 0.001 |  | 2009-2017 | 0.59 (0.58 to 0.60) | < 0.001 |  | 2017-2019 | 0.34 (0.26 to 0.42) | < 0.001 |
| Prevalence | 0.34 (0.33 to 0.35) | < 0.001 |  | 1990-1996 | 0.32 (0.31 to 0.33) | < 0.001 |  | 1996-1999 | 0.38 (0.35 to 0.42) | < 0.001 |  | 1999-2011 | 0.28 (0.28 to 0.29) | < 0.001 |  | 2011-2014 | 0.62 (0.58 to 0.65) | < 0.001 |  | 2014-2017 | 0.17 (0.14 to 0.21) | < 0.001 |  | 2017-2019 | -0.14 (-0.17 to -0.10) | < 0.001 |
| Mortality | 0.45 (0.39 to 0.51) | < 0.001 |  | 1990-1995 | 0.36 (0.19 to 0.54) | < 0.001 |  | 1995-2002 | 1.38 (1.25 to 1.50) | < 0.001 |  | 2002-2015 | 0.25 (0.21 to 0.30) | < 0.001 |  | 2015-2019 | -0.39 (-0.69 to -0.08) | 0.016 |  | - | - | - |  | - | - | - |
| DALYs | 0.24 (0.16 to 0.31) | < 0.001 |  | 1990-1995 | 0.15 (0.01 to 0.29) | 0.038 |  | 1995-1999 | 1.03 (0.74 to 1.33) | < 0.001 |  | 1999-2003 | 0.47 (0.17 to 0.76) | 0.004 |  | 2003-2013 | -0.13 (-0.18 to -0.07) | < 0.001 |  | 2013-2017 | 0.36 (0.02 to 0.71) | 0.039 |  | 2019-2019 | -1.01 (-1.76 to -0.26) | 0.012 |

*Age-standardized rates were standardized with GBD 2019 world population age standard.

^a^The *P*-values were calculated using the Monte Carlo Permutation method.

CKD, chronic kidney disease; GBD, Global Burden of Disease Study; DALYs, disability-adjusted life years; AAPC, average annual percentage change; APC, annual percent change; CI, confidence interval.

**Table S2. Changes in incident cases** according to population-level determinants from 1990 to 2019 globally and by SDI levels as well as WHO regions.

|  | Change due to population-level determinants (% contribution to the total changes) | | | | | | |
| --- | --- | --- | --- | --- | --- | --- | --- |
| Location | Overall different |  | Population growth |  | Aging |  | Epidemiological change |
| Global | 11,190,575.09 |  | 4,623,014.73 (41.31%) |  | 4,062,898.56 (36.31%) |  | 2,504,661.80 (22.38%) |
| High SDI | 2,515,611.95 |  | 788,979.47 (31.36%) |  | 1,317,277.08 (52.36%) |  | 409,355.40 (16.27%) |
| High-middle SDI | 2,352,164.37 |  | 612,051.29 (26.02%) |  | 1,096,555.40 (46.62%) |  | 643,557.68 (27.36%) |
| Middle SDI | 3,919,422.78 |  | 1,190,372.69 (30.37%) |  | 1,629,060.00 (41.56%) |  | 1,099,990.09 (28.07%) |
| Low-middle SDI | 1,759,392.00 |  | 789,158.64 (44.85%) |  | 510,651.74 (29.02%) |  | 459,581.63 (26.12%) |
| Low SDI | 636,710.28 |  | 478,723.30 (75.19%) |  | 1,027.43 (0.16%) |  | 156,959.55 (24.65%) |
| Region of the Americas | 2,320,868.15 |  | 914,778.83 (39.42%) |  | 928,571.13 (40.01%) |  | 477,518.19 (20.57%) |
| African Region | 686,840.56 |  | 497,302.75 (72.40%) |  | -7,972.65 (-1.16%) |  | 197,510.46 (28.76%) |
| South-East Asia Region | 2,121,007.45 |  | 908,477.20 (42.83%) |  | 764,538.47 (36.05%) |  | 447,991.77 (21.12%) |
| Eastern Mediterranean Region | 1,272,328.61 |  | 634,005.95 (49.83%) |  | 156,600.58 (12.31%) |  | 481,722.08 (37.86%) |
| Western Pacific Region | 3,048,679.76 |  | 720,196.71 (23.62%) |  | 1,993,918.16 (65.4%) |  | 334,564.89 (10.97%) |
| European Region | 1,696,678.58 |  | 219,241.05 (12.92%) |  | 797,074.91 (46.98%) |  | 680,362.62 (40.10%) |

SDI, sociodemographic index; WHO, World Health Organization.

**Table S3.** Changes in DALYs according to cause of CKD from 1990 to 2019 globally and by SDI levels as well as WHO regions.

|  | Change due to population-level determinants (% contribution to the total changes) | | | | | | | | | | | | | | |
| --- | --- | --- | --- | --- | --- | --- | --- | --- | --- | --- | --- | --- | --- | --- | --- |
| Location | Overall different |  | Population growth |  | Aging |  | Glomerulonephritis |  | Other causes |  | Hypertension |  | T1DM |  | T2DM |
| Global | 20,830,422.06 |  | 13,164,020.19 (63.20%) |  | 5,115,644.08 (24.56%) |  | 286,922.82 (1.38%) |  | 607,289.70 (2.92%) |  | 653,391.41 (3.14%) |  | -42,204.00 (-0.20%) |  | 1,045,357.88 (5.02%) |
| High SDI | 2,854,221.54 |  | 1,002,567.67 (35.13%) |  | 1,088,333.40 (38.13%) |  | 37,028.16 (1.30%) |  | 110,513.19 (3.87%) |  | 256,703.32 (8.99%) |  | 25,355.53 (0.89%) |  | 333,720.26 (11.69%) |
| High-middle SDI | 2,330,444.49 |  | 1,570,010.55 (67.37%) |  | 1,142,853.29 (49.04%) |  | -161,456.98 (-6.93%) |  | -138,643.38 (-5.95%) |  | -7,819.18 (-0.34%) |  | -87,461.68 (-3.75%) |  | 12,961.86 (0.56%) |
| Middle SDI | 8,348,497.84 |  | 4,922,358.32 (58.96%) |  | 2,859,515.48 (34.25%) |  | 106,593.28 (1.28%) |  | 192,938.33 (2.31%) |  | 133,812.41 (1.60%) |  | -112,382.99 (-1.35%) |  | 245,663 (2.94%) |
| Low-middle SDI | 5,131,321.30 |  | 3,881,679.18 (75.65%) |  | 949,212.17 (18.5%) |  | 50,526.41 (0.98%) |  | 88,368.89 (1.72%) |  | 2,798.37 (0.05%) |  | 11,061.54 (0.22%) |  | 147,674.74 (2.88%) |
| Low SDI | 2,149,887.57 |  | 2,350,261.73 (109.32%) |  | -47,319.08 (-2.20%) |  | -68,356.34 (-3.18%) |  | 13,731.20 (0.64%) |  | -78,014.23 (-3.63%) |  | -5,708.64 (-0.27%) |  | -14,707.07 (-0.68%) |
| Region of the Americas | 5,383,858.68 |  | 2,202,959.61 (40.92%) |  | 1,054,531.79 (19.59%) |  | 373,096.49 (6.93%) |  | 441,798.75 (8.21%) |  | 510,750.48 (9.49%) |  | 137,132.81 (2.55%) |  | 663,588.74 (12.33%) |
| African Region | 2,004,758.04 |  | 2,321,292.88 (115.79%) |  | -52,102.10 (-2.60%) |  | -107,087.17 (-5.34%) |  | -20,675.54 (-1.03%) |  | -79,312.17 (-3.96%) |  | -28,960.54 (-1.44%) |  | -28,397.32 (-1.42%) |
| South-East Asia Region | 5,656,758.63 |  | 4,689,800.80 (82.91%) |  | 1,466,095.00 (25.92%) |  | -203,759.39 (-3.60%) |  | -88,453.77 (-1.56%) |  | -170,518.76 (-3.01%) |  | -88,174.38 (-1.56%) |  | 51,769.13 (0.92%) |
| Eastern Mediterranean Region | 2,584,425.42 |  | 2,074,415.70 (80.27%) |  | 107,092.16 (4.14%) |  | 89,907.09 (3.48%) |  | 127,009.20 (4.91%) |  | 63,613.71 (2.46%) |  | 36,508.60 (1.41%) |  | 85,878.95 (3.32%) |
| Western Pacific Region | 3,924,051.26 |  | 2,437,450.01 (62.12%) |  | 2,503,025.28 (63.79%) |  | -173,364.13 (-4.42%) |  | -251,801.9 (-6.42%) |  | -158,088.63 (-4.03%) |  | -215,963.17 (-5.50%) |  | -217,206.21 (-5.54%) |
| European Region | 1,208,348.10 |  | 423,811.11 (35.07%) |  | 651,372.35 (53.91%) |  | -41,022.33 (-3.39%) |  | 38,278.55 (3.17%) |  | 73,551.72 (6.09%) |  | -8,350.76 (-0.69%) |  | 70,707.46 (5.85%) |

DALYs, disability-adjusted life-years; CKD, chronic kidney disease; SDI, social-demographic index; WHO, World Health Organization; T1DM, type 1 diabetes mellitus; T2DM type 2 diabetes mellitus.

**Table S4. Frontier DALYs, and effective** difference by country or territory.

| Location | SDI | Age-standardized DALYs | Frontier DALYs | Effective difference | Effective difference rank (Age-standardized DALY rank) |
| --- | --- | --- | --- | --- | --- |
| Afghanistan | 0.343 | 1,230.37 (958.38 to 1,680.46) | 399.95 | 830.42 | 154 (178) |
| Albania | 0.681 | 278.25 (221.25 to 342.24) | 85.79 | 192.45 | 51 (42) |
| Algeria | 0.652 | 701.53 (567.99 to 889.89) | 88.63 | 612.90 | 127 (107) |
| American Samoa | 0.712 | 1,596.63 (1,364.49 to 1,879.91) | 86.45 | 1,510.18 | 196 (196) |
| Andorra | 0.894 | 186.21 (149.66 to 225.42) | 86.42 | 99.80 | 21 (20) |
| Angola | 0.470 | 609.26 (440.86 to 763.81) | 156.59 | 452.67 | 101 (86) |
| Antigua and Barbuda | 0.743 | 1,032.36 (888.45 to 1,185.40) | 86.08 | 946.28 | 166 (162) |
| Argentina | 0.708 | 605.35 (559.42 to 653.29) | 86.13 | 519.22 | 113 (83) |
| Armenia | 0.689 | 316.39 (269.98 to 362.87) | 86.65 | 229.74 | 59 (49) |
| Australia | 0.839 | 208.50 (184.75 to 233.22) | 86.00 | 122.49 | 29 (27) |
| Austria | 0.849 | 249.37 (223.19 to 276.75) | 86.05 | 163.32 | 43 (38) |
| Azerbaijan | 0.683 | 536.69 (462.31 to 619.25) | 85.96 | 450.73 | 100 (72) |
| Bahamas | 0.796 | 933.35 (780.77 to 1,119.96) | 86.77 | 846.58 | 158 (154) |
| Bahrain | 0.751 | 711.89 (588.82 to 844.68) | 86.07 | 625.83 | 129 (109) |
| Bangladesh | 0.483 | 344.51 (288.14 to 407.74) | 155.40 | 189.10 | 49 (51) |
| Barbados | 0.742 | 744.54 (628.36 to 875.69) | 85.95 | 658.58 | 133 (115) |
| Belarus | 0.745 | 128.92 (104.56 to 156.45) | 86.40 | 42.51 | 5 (4) |
| Belgium | 0.851 | 190.31 (167.14 to 214.27) | 86.31 | 104.00 | 22 (22) |
| Belize | 0.603 | 1,214.44 (1,059.15 to 1,393.09) | 88.46 | 1,125.98 | 180 (175) |
| Benin | 0.352 | 824.78 (630.43 to 1,067.96) | 400.36 | 424.41 | 95 (138) |
| Bermuda | 0.813 | 446.74 (376.28 to 529.67) | 85.85 | 360.89 | 85 (63) |
| Bhutan | 0.455 | 741.37 (555.09 to 934.40) | 182.89 | 558.48 | 114 (114) |
| Bolivia (Plurinational State of) | 0.566 | 1,227.15 (956.53 to 1,537.15) | 132.32 | 1,094.83 | 177 (177) |
| Bosnia and Herzegovina | 0.718 | 307.57 (255.56 to 367.75) | 85.59 | 221.98 | 57 (46) |
| Botswana | 0.634 | 932.50 (673.13 to 1,233.63) | 88.60 | 843.90 | 157 (153) |
| Brazil | 0.640 | 504.23 (465.82 to 542.52) | 88.59 | 415.64 | 93 (69) |
| Brunei Darussalam | 0.823 | 769.61 (686.72 to 851.71) | 86.04 | 683.58 | 142 (124) |
| Bulgaria | 0.764 | 440.69 (360.89 to 536.55) | 86.54 | 354.15 | 83 (62) |
| Burkina Faso | 0.257 | 799.55 (651.75 to 952.28) | 556.72 | 242.84 | 64 (131) |
| Burundi | 0.284 | 620.20 (489.59 to 775.78) | 476.62 | 143.59 | 36 (91) |
| Cabo Verde | 0.525 | 572.04 (497.93 to 647.97) | 155.32 | 416.71 | 94 (76) |
| Cambodia | 0.469 | 608.40 (510.70 to 714.18) | 176.46 | 431.95 | 96 (85) |
| Cameroon | 0.490 | 1,076.47 (817.47 to 1,393.59) | 157.22 | 919.26 | 162 (166) |
| Canada | 0.873 | 209.41 (186.10 to 236.51) | 86.70 | 122.72 | 30 (28) |
| Central African Republic | 0.274 | 840.67 (634.11 to 1,094.27) | 506.44 | 334.23 | 79 (141) |
| Chad | 0.238 | 810.14 (618.31 to 1,045.62) | 557.86 | 252.27 | 65 (135) |
| Chile | 0.759 | 436.30 (398.82 to 476.07) | 86.88 | 349.43 | 81 (60) |
| China | 0.686 | 311.64 (267.99 to 353.67) | 86.20 | 225.44 | 58 (47) |
| Colombia | 0.633 | 501.42 (407.07 to 617.49) | 88.46 | 412.96 | 91 (68) |
| Comoros | 0.455 | 630.56 (506.43 to 764.34) | 181.47 | 449.19 | 99 (92) |
| Congo | 0.568 | 750.72 (535.86 to 971.33) | 143.13 | 607.58 | 126 (117) |
| Cook Islands | 0.764 | 850.50 (706.15 to 1,010.20) | 85.74 | 764.75 | 152 (145) |
| Costa Rica | 0.680 | 766.13 (617.11 to 935.90) | 85.82 | 680.31 | 141 (123) |
| Croatia | 0.794 | 235.60 (196.58 to 280.59) | 85.88 | 149.72 | 38 (33) |
| Cuba | 0.668 | 471.92 (394.86 to 561.77) | 85.48 | 386.45 | 87 (64) |
| Cyprus | 0.841 | 315.69 (278.65 to 353.32) | 85.38 | 230.32 | 60 (48) |
| Czechia | 0.828 | 174.41 (145.34 to 206.50) | 85.75 | 88.66 | 16 (15) |
| Democratic People's Republic of Korea | 0.558 | 487.81 (399.40 to 587.41) | 139.61 | 348.21 | 80 (66) |
| Democratic Republic of the Congo | 0.382 | 612.61 (496.50 to 743.01) | 403.35 | 209.26 | 56 (89) |
| Denmark | 0.890 | 191.94 (170.15 to 215.01) | 86.40 | 105.54 | 24 (23) |
| Djibouti | 0.459 | 675.87 (527.45 to 888.01) | 183.03 | 492.84 | 109 (102) |
| Dominica | 0.729 | 1,336.71 (1,108.43 to 1,619.83) | 85.99 | 1,250.72 | 187 (186) |
| Dominican Republic | 0.592 | 759.53 (585.72 to 979.84) | 92.04 | 667.50 | 136 (120) |
| Ecuador | 0.640 | 1,109.00 (899.22 to 1,381.37) | 88.60 | 1,020.4 | 170 (167) |
| Egypt | 0.658 | 1,009.46 (660.38 to 1,386.31) | 87.59 | 921.87 | 163 (159) |
| El Salvador | 0.573 | 1,910.95 (1,484.58 to 2,461.33) | 140.54 | 1,770.41 | 199 (200) |
| Equatorial Guinea | 0.685 | 720.98 (526.26 to 981.01) | 86.23 | 634.75 | 130 (111) |
| Eritrea | 0.396 | 701.57 (504.19 to 941.09) | 400.90 | 300.67 | 76 (108) |
| Estonia | 0.835 | 260.90 (215.24 to 312.70) | 85.67 | 175.23 | 46 (40) |
| Eswatini | 0.577 | 1,314.95 (959.32 to 1,698.25) | 141.60 | 1,173.34 | 181 (185) |
| Ethiopia | 0.343 | 592.99 (523.05 to 674.12) | 401.68 | 191.31 | 50 (81) |
| Fiji | 0.664 | 1,287.88 (1,048.66 to 1,570.46) | 88.45 | 1,199.43 | 184 (183) |
| Finland | 0.856 | 111.94 (97.61 to 129.02) | 85.29 | 26.65 | 2 (1) |
| France | 0.834 | 139.62 (120.94 to 160.04) | 86.68 | 52.94 | 7 (7) |
| Gabon | 0.656 | 929.74 (627.29 to 1,198.08) | 88.66 | 841.08 | 156 (152) |
| Gambia | 0.399 | 807.52 (635.46 to 1,007.36) | 400.05 | 407.48 | 90 (133) |
| Georgia | 0.702 | 393.33 (336.58 to 451.55) | 86.18 | 307.15 | 77 (57) |
| Germany | 0.898 | 241.74 (216.70 to 266.90) | 85.95 | 155.79 | 40 (35) |
| Ghana | 0.557 | 798.23 (597.45 to 1,002.46) | 136.41 | 661.82 | 134 (130) |
| Greece | 0.794 | 289.40 (261.51 to 317.38) | 86.99 | 202.41 | 52 (43) |
| Greenland | 0.761 | 295.61 (249.11 to 343.91) | 86.45 | 209.16 | 55 (44) |
| Grenada | 0.669 | 1,337.12 (1,186.74 to 1,485.69) | 87.08 | 1,250.03 | 186 (187) |
| Guam | 0.813 | 942.55 (810.56 to 1,107.41) | 86.04 | 856.51 | 159 (155) |
| Guatemala | 0.526 | 1,498.59 (1,213.21 to 1,841.56) | 155.14 | 1,343.46 | 193 (194) |
| Guinea | 0.325 | 899.45 (710.76 to 1,120.95) | 401.72 | 497.73 | 110 (149) |
| Guinea-Bissau | 0.355 | 1,076.07 (855.94 to 1,324.91) | 400.92 | 675.15 | 138 (165) |
| Guyana | 0.618 | 1,415.11 (1,133.41 to 1,756.76) | 88.50 | 1,326.61 | 192 (192) |
| Haiti | 0.432 | 1,043.24 (770.41 to 1,508.47) | 398.04 | 645.19 | 131 (163) |
| Honduras | 0.496 | 1,407.49 (1,152.53 to 1,787.96) | 155.67 | 1251.82 | 189 (191) |
| Hungary | 0.791 | 225.32 (190.93 to 266.10) | 85.83 | 139.49 | 35 (32) |
| Iceland | 0.869 | 114.27 (98.22 to 131.40) | 86.25 | 28.02 | 3 (2) |
| India | 0.566 | 612.57 (535.56 to 691.20) | 140.38 | 472.19 | 103 (88) |
| Indonesia | 0.660 | 673.41 (588.41 to 764.67) | 88.34 | 585.07 | 122 (101) |
| Iran (Islamic Republic of) | 0.670 | 486.68 (443.93 to 530.46) | 86.51 | 400.18 | 88 (65) |
| Iraq | 0.671 | 991.20 (797.31 to 1,215.04) | 86.24 | 904.96 | 160 (157) |
| Ireland | 0.867 | 177.65 (154.56 to 202.23) | 85.78 | 91.87 | 19 (18) |
| Israel | 0.803 | 381.31 (345.06 to 419.21) | 85.82 | 295.49 | 74 (55) |
| Italy | 0.801 | 171.33 (151.41 to 192.52) | 86.00 | 85.34 | 15 (14) |
| Jamaica | 0.684 | 918.54 (748.81 to 1,125.74) | 86.26 | 832.28 | 155 (151) |
| Japan | 0.870 | 221.90 (192.44 to 250.54) | 84.99 | 136.92 | 33 (30) |
| Jordan | 0.731 | 825.82 (707.85 to 957.14) | 85.71 | 740.11 | 149 (139) |
| Kazakhstan | 0.723 | 381.86 (336.00 to 431.67) | 87.02 | 294.84 | 73 (56) |
| Kenya | 0.508 | 587.44 (500.48 to 687.26) | 152.92 | 434.52 | 97 (79) |
| Kiribati | 0.527 | 1,706.49 (1,308.14 to 2,159.50) | 155.26 | 1,551.22 | 197 (197) |
| Kuwait | 0.851 | 418.09 (349.86 to 488.74) | 85.20 | 332.89 | 78 (59) |
| Kyrgyzstan | 0.596 | 399.32 (349.69 to 454.67) | 104.78 | 294.53 | 72 (58) |
| Lao People's Democratic Republic | 0.490 | 1,154.54 (913.57 to 1,438.63) | 153.28 | 1,001.26 | 169 (170) |
| Latvia | 0.820 | 200.31 (169.34 to 238.15) | 87.01 | 113.30 | 26 (25) |
| Lebanon | 0.708 | 574.66 (449.11 to 717.08) | 86.18 | 488.48 | 108 (77) |
| Lesotho | 0.507 | 1,246.29 (893.24 to 1,617.35) | 156.04 | 1,090.25 | 176 (179) |
| Liberia | 0.370 | 815.14 (610.14 to 1,095.49) | 400.04 | 415.11 | 92 (137) |
| Libya | 0.709 | 755.70 (552.53 to 970.69) | 84.75 | 670.95 | 137 (118) |
| Lithuania | 0.843 | 156.23 (131.15 to 183.27) | 86.07 | 70.16 | 12 (11) |
| Luxembourg | 0.895 | 198.39 (173.00 to 229.21) | 85.83 | 112.55 | 25 (24) |
| Madagascar | 0.396 | 514.70 (408.65 to 659.13) | 398.92 | 115.78 | 27 (70) |
| Malawi | 0.384 | 642.77 (534.27 to 761.07) | 400.07 | 242.70 | 63 (94) |
| Malaysia | 0.737 | 690.72 (575.96 to 828.12) | 86.33 | 604.39 | 125 (105) |
| Maldives | 0.562 | 773.83 (659.66 to 893.21) | 125.51 | 648.32 | 132 (125) |
| Mali | 0.263 | 810.65 (635.31 to 1,032.21) | 537.23 | 273.42 | 69 (136) |
| Malta | 0.801 | 224.29 (196.19 to 255.88) | 86.42 | 137.87 | 34 (31) |
| Marshall Islands | 0.544 | 1,533.75 (1,163.87 to 2,020.13) | 142.91 | 1,390.85 | 195 (195) |
| Mauritania | 0.496 | 727.57 (545.42 to 937.31) | 154.27 | 573.30 | 119 (112) |
| Mauritius | 0.705 | 2,032.48 (1,683.03 to 2,441.10) | 85.70 | 1,946.78 | 202 (202) |
| Mexico | 0.649 | 1,765.46 (1,544.91 to 2,006.16) | 88.49 | 1,676.97 | 198 (198) |
| Micronesia (Federated States of) | 0.580 | 2,162.73 (1,584.61 to 2,761.55) | 128.99 | 2,033.74 | 204 (204) |
| Monaco | 0.902 | 143.17 (119.81 to 167.20) | 86.13 | 57.04 | 10 (9) |
| Mongolia | 0.606 | 563.52 (460.67 to 689.92) | 88.60 | 474.92 | 104 (74) |
| Montenegro | 0.791 | 366.38 (316.23 to 425.05) | 86.24 | 280.14 | 71 (54) |
| Morocco | 0.548 | 844.57 (673.16 to 1,035.89) | 139.65 | 704.92 | 147 (143) |
| Mozambique | 0.307 | 649.27 (520.54 to 795.58) | 416.94 | 232.33 | 61 (95) |
| Myanmar | 0.521 | 863.75 (719.80 to 1,035.50) | 163.17 | 700.58 | 144 (146) |
| Namibia | 0.612 | 659.69 (486.03 to 888.71) | 88.65 | 571.03 | 118 (98) |
| Nauru | 0.618 | 1,905.20 (1,405.14 to 2,364.18) | 88.44 | 1,816.76 | 200 (199) |
| Nepal | 0.422 | 697.49 (522.11 to 897.63) | 399.59 | 297.90 | 75 (106) |
| Netherlands | 0.883 | 177.60 (156.83 to 198.52) | 85.80 | 91.80 | 18 (17) |
| New Zealand | 0.840 | 244.89 (220.65 to 269.54) | 85.43 | 159.46 | 42 (37) |
| Nicaragua | 0.517 | 2,111.98 (1,753.72 to 2,500.72) | 153.71 | 1,958.27 | 203 (203) |
| Niger | 0.162 | 741.00 (567.80 to 950.58) | 573.84 | 167.17 | 44 (113) |
| Nigeria | 0.515 | 612.67 (495.43 to 755.18) | 153.48 | 459.19 | 102 (90) |
| Niue | 0.711 | 1,337.91 (1,008.92 to 1,688.39) | 86.13 | 1,251.78 | 188 (188) |
| North Macedonia | 0.744 | 352.21 (292.29 to 425.00) | 86.68 | 265.53 | 67 (52) |
| Northern Mariana Islands | 0.771 | 1,473.42 (1,257.07 to 1,717.32) | 86.29 | 1,387.13 | 194 (193) |
| Norway | 0.913 | 139.75 (123.31 to 158.48) | 85.48 | 54.27 | 9 (8) |
| Oman | 0.783 | 491.30 (428.61 to 558.94) | 85.54 | 405.76 | 89 (67) |
| Pakistan | 0.449 | 1,156.35 (873.19 to 1,447.54) | 185.20 | 971.14 | 167 (171) |
| Palau | 0.738 | 1,956.22 (1,574.39 to 2,372.11) | 85.00 | 1,871.22 | 201 (201) |
| Palestine | 0.588 | 809.92 (707.33 to 918.09) | 108.23 | 701.69 | 146 (134) |
| Panama | 0.686 | 787.46 (632.46 to 964.62) | 86.08 | 701.38 | 145 (129) |
| Papua New Guinea | 0.394 | 528.91 (425.10 to 653.43) | 403.69 | 125.23 | 31 (71) |
| Paraguay | 0.638 | 894.27 (707.99 to 1,124.94) | 88.66 | 805.61 | 153 (148) |
| Peru | 0.648 | 603.42 (470.40 to 766.59) | 88.60 | 514.82 | 112 (82) |
| Philippines | 0.623 | 1,279.50 (1,090.34 to 1,488.44) | 88.52 | 1,190.98 | 183 (182) |
| Poland | 0.802 | 189.96 (161.71 to 220.86) | 85.78 | 104.18 | 23 (21) |
| Portugal | 0.743 | 244.38 (219.08 to 271.23) | 85.76 | 158.62 | 41 (36) |
| Puerto Rico | 0.814 | 774.32 (631.47 to 946.37) | 86.19 | 688.13 | 143 (127) |
| Qatar | 0.830 | 803.55 (662.78 to 963.84) | 86.16 | 717.39 | 148 (132) |
| Republic of Korea | 0.878 | 202.96 (182.77 to 224.50) | 86.82 | 116.14 | 28 (26) |
| Republic of Moldova | 0.696 | 174.44 (152.02 to 199.25) | 85.41 | 89.03 | 17 (16) |
| Romania | 0.760 | 259.68 (220.58 to 304.53) | 85.93 | 173.75 | 45 (39) |
| Russian Federation | 0.805 | 213.78 (183.68 to 245.92) | 86.22 | 127.56 | 32 (29) |
| Rwanda | 0.429 | 585.80 (469.84 to 712.56) | 400.43 | 185.37 | 48 (78) |
| Saint Kitts and Nevis | 0.746 | 1,388.05 (1,114.01 to 1,660.99) | 85.38 | 1,302.67 | 191 (189) |
| Saint Lucia | 0.670 | 998.50 (856.75 to 1165.82) | 85.56 | 912.94 | 161 (158) |
| Saint Vincent and the Grenadines | 0.627 | 1,061.06 (917.51 to 1,225.69) | 88.45 | 972.61 | 168 (164) |
| Samoa | 0.641 | 1,262.68 (1,021.20 to 1,546.64) | 88.69 | 1,173.99 | 182 (181) |
| San Marino | 0.884 | 119.53 (91.33 to 152.48) | 85.21 | 34.32 | 4 (3) |
| Sao Tome and Principe | 0.502 | 1,218.56 (983.37 to 1,449.41) | 160.11 | 1,058.45 | 173 (176) |
| Saudi Arabia | 0.805 | 1,197.72 (982.79 to 1,421.32) | 85.67 | 1,112.05 | 179 (174) |
| Senegal | 0.389 | 842.15 (643.58 to 1,100.46) | 400.00 | 442.15 | 98 (142) |
| Serbia | 0.767 | 359.29 (300.31 to 423.70) | 85.82 | 273.47 | 70 (53) |
| Seychelles | 0.724 | 1,123.45 (994.24 to 1,257.61) | 86.47 | 1,036.97 | 171 (168) |
| Sierra Leone | 0.347 | 756.46 (584.94 to 965.44) | 400.00 | 356.46 | 84 (119) |
| Singapore | 0.861 | 269.29 (241.71 to 297.89) | 84.74 | 184.55 | 47 (41) |
| Slovakia | 0.812 | 237.73 (196.59 to 285.45) | 86.46 | 151.27 | 39 (34) |
| Slovenia | 0.840 | 139.14 (112.74 to 168.69) | 85.66 | 53.47 | 8 (6) |
| Solomon Islands | 0.407 | 975.87 (812.34 to 1,155.90) | 400.70 | 575.17 | 120 (156) |
| Somalia | 0.081 | 762.10 (593.39 to 984.14) | 762.10 | 0.00 | 1 (121) |
| South Africa | 0.678 | 765.11 (706.48 to 825.38) | 86.10 | 679.00 | 140 (122) |
| South Sudan | 0.363 | 641.58 (477.47 to 838.84) | 400.54 | 241.04 | 62 (93) |
| Spain | 0.767 | 180.18 (161.20 to 200.20) | 85.76 | 94.42 | 20 (19) |
| Sri Lanka | 0.690 | 679.34 (540.26 to 854.06) | 86.01 | 593.33 | 123 (103) |
| Sudan | 0.515 | 717.30 (535.03 to 1,013.09) | 153.36 | 563.95 | 116 (110) |
| Suriname | 0.636 | 1,296.21 (1,108.67 to 1,518.53) | 88.63 | 1,207.58 | 185 (184) |
| Sweden | 0.872 | 145.37 (128.90 to 164.63) | 86.41 | 58.96 | 11 (10) |
| Switzerland | 0.929 | 163.44 (142.64 to 185.48) | 85.36 | 78.08 | 14 (13) |
| Syrian Arab Republic | 0.619 | 684.86 (556.83 to 836.76) | 88.62 | 596.23 | 124 (104) |
| Taiwan (Province of China) | 0.868 | 590.40 (490.64 to 701.23) | 86.52 | 503.88 | 111 (80) |
| Tajikistan | 0.539 | 298.16 (252.87 to 350.40) | 152.37 | 145.79 | 37 (45) |
| Thailand | 0.687 | 747.51 (601.84 to 934.74) | 84.93 | 662.58 | 135 (116) |
| Timor-Leste | 0.514 | 833.00 (612.76 to 1,049.22) | 155.96 | 677.03 | 139 (140) |
| Togo | 0.417 | 776.04 (622.58 to 967.26) | 400.71 | 375.34 | 86 (128) |
| Tokelau | 0.626 | 1,157.87 (895.19 to 1,541.24) | 88.64 | 1,069.23 | 174 (172) |
| Tonga | 0.636 | 1,165.11 (929.05 to 1,507.65) | 88.65 | 1,076.46 | 175 (173) |
| Trinidad and Tobago | 0.757 | 1,013.45 (792.65 to 1,280.19) | 85.89 | 927.56 | 164 (160) |
| Tunisia | 0.672 | 568.57 (441.00 to 718.86) | 85.98 | 482.59 | 107 (75) |
| Turkey | 0.748 | 562.02 (473.61 to 667.56) | 85.91 | 476.11 | 105 (73) |
| Turkmenistan | 0.670 | 651.42 (540.11 to 791.75) | 85.41 | 566.00 | 117 (96) |
| Tuvalu | 0.589 | 1,388.07 (1,054.44 to 1,852.50) | 123.95 | 1,264.12 | 190 (190) |
| Uganda | 0.404 | 607.78 (483.98 to 746.93) | 402.71 | 205.07 | 53 (84) |
| Ukraine | 0.736 | 156.69 (132.82 to 183.41) | 85.82 | 70.87 | 13 (12) |
| United Arab Emirates | 0.880 | 1,136.93 (787.65 to 1,645.06) | 86.25 | 1,050.69 | 172 (169) |
| United Kingdom | 0.847 | 138.20 (119.87 to 159.47) | 86.48 | 51.71 | 6 (5) |
| United Republic of Tanzania | 0.423 | 667.95 (561.52 to 790.01) | 398.60 | 269.36 | 68 (99) |
| United States of America | 0.859 | 438.20 (402.09 to 477.34) | 86.70 | 351.50 | 82 (61) |
| United States Virgin Islands | 0.799 | 848.73 (724.68 to 988.03) | 85.78 | 762.96 | 151 (144) |
| Uruguay | 0.697 | 343.18 (314.17 to 372.73) | 85.75 | 257.43 | 66 (50) |
| Uzbekistan | 0.631 | 670.40 (576.62 to 771.85) | 88.66 | 581.74 | 121 (100) |
| Vanuatu | 0.485 | 1,252.52 (947.51 to 1628.46) | 153.18 | 1,099.34 | 178 (180) |
| Venezuela (Bolivarian Republic of) | 0.607 | 1,020.90 (801.82 to 1297.67) | 88.66 | 932.24 | 165 (161) |
| Viet Nam | 0.617 | 651.90 (527.73 to 779.63) | 88.60 | 563.29 | 115 (97) |
| Yemen | 0.412 | 611.56 (486.80 to 769.32) | 403.94 | 207.61 | 54 (87) |
| Zambia | 0.505 | 774.21 (632.41 to 938.65) | 155.87 | 618.35 | 128 (126) |
| Zimbabwe | 0.476 | 915.70 (683.94 to 1,309.60) | 154.00 | 761.70 | 150 (150) |

DALYs, disability-adjusted life years; SDI, sociodemographic index.

**Table S5. BAPC prediction of incidence of CKD by sex and SDI** in 2020-2030.

|  | 2020 | |  | 2021 | |  | 2022 | |  | 2023 | |  | 2024 | |  | 2025 | |  | 2026 | |  | 2027 | |  | 2028 | |  | 2029 | |  | 2030 | |
| --- | --- | --- | --- | --- | --- | --- | --- | --- | --- | --- | --- | --- | --- | --- | --- | --- | --- | --- | --- | --- | --- | --- | --- | --- | --- | --- | --- | --- | --- | --- | --- | --- |
|  | Rate per 100,000 (95% CI) | Cases No. ×10^4^ (95% CI) |  | Rate per 100,000 (95% CI) | Cases No. ×10^4^ (95% CI) |  | Rate per 100,000 (95% CI) | Cases No. ×10^4^ (95% CI) |  | Rate per 100,000 (95% CI) | Cases No. ×10^4^ (95% CI) |  | Rate per 100,000 (95% CI) | Cases No. ×10^4^ (95% CI) |  | Rate per 100,000 (95% CI) | Cases No. ×10^4^ (95% CI) |  | Rate per 100,000 (95% CI) | Cases No. ×10^4^ (95% CI) |  | Rate per 100,000 (95% CI) | Cases No. ×10^4^ (95% CI) |  | Rate per 100,000 (95% CI) | Cases No. ×10^4^ (95% CI) |  | Rate per 100,000 (95% CI) | Cases No. ×10^4^ (95% CI) |  | Rate per 100,000 (95% CI) | Cases No. ×10^4^ (95% CI) |
| Both sexes |  |  |  |  |  |  |  |  |  |  |  |  |  |  |  |  |  |  |  |  |  |  |  |  |  |  |  |  |  |  |  |  |
| Global | 234.72 (135.40 to 586.10) | 1,962.75 (1,933.26 to 1,992.24) |  | 235.82 (127.07 to 646.31) | 2,022.98 (1,977.51 to 2,068.45) |  | 236.90 (96.74 to 904.14) | 2,083.90 (2,016.97 to 2,150.82) |  | 237.99 (61.31 to 1,324.41) | 2,146.73 (2,053.62 to 2,239.84) |  | 239.10 (35.21 to 1,757.40) | 2,212.32 (2,088.45 to 2,336.19) |  | 240.26 (20.23 to 2,119.06) | 2,279.98 (2,120.77 to 2,439.20) |  | 241.42 (10.77 to 2,399.63) | 2,348.64 (2,149.64 to 2,547.64) |  | 242.60 (5.99 to 2,612.22) | 2,417.57 (2,174.39 to 2,660.76) |  | 243.80 (3.18 to 2,771.67) | 2,478.82 (2,195.88 to 2,779.75) |  | 245.04 (1.70 to 2,889.26) | 2,560.42 (2,214.84 to 2,906.00) |  | 246.36 (0.93 to 2,973.94) | 2,634.66 (2,230.37 to 3,038.95) |
| High SDI | 282.61 (258.93 to 315.40) | 507.71 (498.89 to 516.54) |  | 284.65 (257.27 to 325.90) | 521.35 (507.98 to 534.73) |  | 286.73 (249.92 to 355.14) | 535.09 (515.55 to 554.63) |  | 288.85 (239.78 to 397.62) | 549.00 (521.94 to 576.07) |  | 291.04 (232.09 to 439.68) | 563.23 (527.38 to 599.09) |  | 293.31 (225.83 to 474.85) | 577.67 (531.79 to 623.55) |  | 295.67 (220.71 to 502.85) | 592.17 (535.08 to 649.26) |  | 298.10 (216.62 to 525.16) | 606.53 (537.08 to 675.98) |  | 300.60 (213.35 to 543.32) | 620.85 (537.87 to 703.82) |  | 303.21 (210.31 to 558.51) | 635.29 (537.60 to 732.98) |  | 305.95 (207.33 to 571.63) | 649.75 (536.12 to 763.37) |
| High-middle SDI | 211.9 (186.96 to 243.20) | 224.88 (220.04 to 229.72) |  | 212.60 (185.09 to 249.10) | 229.83 (222.88 to 236.78) |  | 213.27 (178.41 to 268.50) | 234.79 (224.98 to 244.60) |  | 213.95 (168.27 to 302.79) | 239.92 (226.64 to 253.21) |  | 214.69 (158.23 to 342.39) | 245.34 (227.99 to 262.70) |  | 215.45 (149.64 to 378.53) | 250.93 (228.95 to 272.92) |  | 216.16 (143.06 to 408.22) | 256.50 (229.37 to 283.64) |  | 216.84 (137.54 to 431.58) | 261.99 (229.20 to 294.78) |  | 217.56 (132.93 to 449.70) | 267.54 (228.58 to 306.50) |  | 218.33 (129.21 to 463.73) | 273.27 (227.59 to 318.95) |  | 219.14 (125.91 to 474.64) | 279.08 (226.13 to 332.03) |
| Middle  SDI | 237.26 (211.41 to 267.17) | 799.36 (785.47 to 813.26) |  | 238.94 (210.37 to 273.72) | 828.61 (807.96 to 849.27) |  | 240.59 (204.35 to 292.95) | 858.00 (828.14 to 887.86) |  | 242.25 (194.81 to 325.72) | 888.46 (847.23 to 929.69) |  | 243.97 (185.45 to 362.93) | 920.26 (865.52 to 975.00) |  | 245.77 (177.41 to 396.61) | 952.91 (882.52 to 1,023.31) |  | 247.54 (171.92 to 424.45) | 985.72 (897.61 to 1,073.83) |  | 249.29 (167.36 to 446.79) | 1,018.46 (910.62 to 1,126.30) |  | 251.06 (163.57 to 464.63) | 1，051.78 (922.08 to 1,181.48) |  | 252.90 (160.36 to 478.88) | 1,086.10 (932.25 to 1,239.95) |  | 254.83 (157.54 to 490.34) | 1,120.89 (940.53 to 1,301.25) |
| Low- middle  SDI | 195.60 (169.59 to 225.33) | 353.18 (345.63 to 360.73) |  | 195.60 (169.59 to 225.33) | 362.89 (351.81 to 373.97) |  | 195.83 (165.87 to 233.10) | 372.82 (356.95 to 388.69) |  | 196.04 (158.24 to 252.18) | 382.99 (361.27 to 404.70) |  | 196.24 (148.56 to 278.88) | 393.49 (364.93 to 422.04) |  | 196.46 (139.78 to 306.75) | 404.28 (367.92 to 440.64) |  | 196.69 (131.76 to 332.22) | 415.30 (370.19 to 460.41) |  | 196.92 (125.19 to 354.19) | 426.47 (371.66 to 481.28) |  | 197.16 (119.53 to 372.69) | 437.88 (372.40 to 503.35) |  | 197.43 (114.40 to 388.11) | 449.65 (372.48 to 526.82) |  | 197.74 (109.73 to 400.96) | 461.77 (371.85 to 551.70) |
| Low SDI | 161.57 (138.58 to 191.02) | 85.08 (83.74 to 86.41) |  | 162.61 (136.67 to 198.06) | 88.38  (86.18 to 90.57) |  | 163.65 (128.22 to 223.42) | 91.78 (88.43 to 95.12) |  | 164.68 (118.41 to 259.35) | 95.32 (90.56 to 100.08) |  | 165.70 (110.61 to 293.32) | 99.05  (92.61 to 105.49) |  | 166.73 (104.60 to 320.80) | 102.96 (94.57 to 111.35) |  | 167.79 (100.25 to 342.20) | 107.03  (96.40 to 117.65) |  | 168.88 (96.88 to 358.94) | 111.23 (98.09 to 124.37) |  | 169.97 (94.26 to 372.21) | 115.60  (99.64 to 131.57) |  | 171.08 (92.27 to 382.88) | 120.19  (101.07 to 139.31) |  | 172.22 (90.48 to 391.54) | 124.99  (102.36 to 147.62) |
| Male |  |  |  |  |  |  |  |  |  |  |  |  |  |  |  |  |  |  |  |  |  |  |  |  |  |  |  |  |  |  |  |  |
| Global | 224.37 (220.98 to 227.76) | 881.43 (867.91 to 894.96) |  | 225.80 (220.69 to 230.92) | 910.05 (889.28 to 930.82) |  | 227.24 (219.88 to 234.59) | 938.94 (908.42 to 969.45) |  | 228.69 (218.69 to 238.68) | 968.72 (926.28 to 1,011.16) |  | 230.17 (217.19 to 243.16) | 999.87 (943.39 to 1,056.35) |  | 231.70 (215.40 to 247.99) | 1,032.06 (959.41 to 1,104.70) |  | 233.23 (213.33 to 253.13) | 1,064.78 (973.88 to 1,155.67) |  | 234.76 (210.99 to 258.53) | 1,097.65 (986.46 to 1,208.84) |  | 236.28 (208.38 to 264.19) | 1,131.19 (997.56 to 1,264.82) |  | 237.84 (205.54 to 270.14) | 1,165.93 (1,007.54 to 1,324.31) |  | 239.44 (202.48 to 276.41) | 1,201.57 (1,016.04 to 1,387.10) |
| High SDI | 273.86 (269.23 to 278.49) | 224.01 (220.09 to 227.93) |  | 275.87 (269.01 to 282.74) | 230.52 (224.68 to 236.36) |  | 277.91 (268.09 to 287.73) | 237.03 (228.58 to 245.48) |  | 280.00 (266.68 to 293.32) | 243.60 (231.94 to 255.25) |  | 282.15 (264.84 to 299.45) | 250.33 (234.93 to 265.74) |  | 284.37 (262.64 to 306.10) | 257.17 (237.47 to 276.87) |  | 286.64 (260.08 to 313.21) | 264.02 (239.51 to 288.53) |  | 288.95 (257.17 to 320.74) | 270.79 (240.97 to 300.61) |  | 291.31 (253.94 to 328.69) | 277.55 (241.91 to 313.19) |  | 293.77 (250.42 to 337.12) | 284.41 (242.41 to 326.40) |  | 296.34 (246.64 to 346.05) | 291.32 (242.43 to 340.21) |
| High-middle SDI | 203.35 (199.22 to 207.48) | 92.34 (90.42 to 94.26) |  | 204.40 (198.44 to 210.37) | 94.74 (91.94 to 97.54) |  | 205.43 (197.05 to 213.82) | 97.15 (93.16 to 101.15) |  | 206.48 (195.24 to 217.73) | 99.65 (94.20 to 105.11) |  | 207.56 (193.07 to 222.06) | 102.31 (95.14 to 109.47) |  | 208.66 (190.57 to 226.75) | 105.05 (95.93 to 114.18) |  | 209.69 (187.71 to 231.68) | 107.81 (96.49 to 119.13) |  | 210.69 (184.53 to 236.86) | 110.53 (96.80 to 124.27) |  | 211.70 (181.09 to 242.31) | 113.31 (96.91 to 129.70) |  | 212.72 (177.39 to 248.05) | 116.19 (96.88 to 135.50) |  | 213.75 (173.43 to 254.06) | 119.13 (96.65 to 141.60) |
| Middle  SDI | 222.39 (218.18 to 226.59) | 359.75 (352.80 to 366.71) |  | 224.52 (218.57 to 230.48) | 373.22 (363.19 to 383.24) |  | 226.66 (218.38 to 234.94) | 386.77 (372.53 to 401.01) |  | 228.80 (217.74 to 239.86) | 400.83 (381.36 to 420.29) |  | 230.98 (216.74 to 245.22) | 415.50 (389.82 to 441.19) |  | 233.22 (215.43 to 251.00) | 430.61 (397.70 to 463.51) |  | 235.46 (213.79 to 257.13) | 445.89 (404.80 to 486.98) |  | 237.69 (211.83 to 263.55) | 461.24 (411.02 to 511.46) |  | 239.91 (209.56 to 270.26) | 476.90 (416.54 to 537.26) |  | 242.16 (207.02 to 277.30) | 493.05 (421.47 to 564.62) |  | 244.46 (204.21 to 284.71) | 509.46 (425.56 to 593.36) |
| Low- middle  SDI | 190.50 (186.19 to 194.82) | 168.03 (164.25 to 171.81) |  | 190.87 (184.85 to 196.89) | 172.70 (167.27 to 178.13) |  | 191.21 (182.94 to 199.49) | 177.48 (169.81 to 185.15) |  | 191.53 (180.58 to 202.48) | 182.38 (171.95 to 192.80) |  | 191.84 (177.88 to 205.81) | 187.43 (173.79 to 201.07) |  | 192.16 (174.88 to 209.43) | 192.64 (175.32 to 209.96) |  | 192.47 (171.62 to 213.32) | 197.96 (176.51 to 219.40) |  | 192.76 (168.10 to 217.43) | 203.36 (177.34 to 229.38) |  | 193.04 (164.34 to 221.75) | 208.89 (177.83 to 239.94) |  | 193.32 (160.36 to 226.28) | 214.61 (178.02 to 251.19) |  | 193.61 (156.19 to 231.04) | 220.50 (177.88 to 263.13) |
| Low SDI | 152.79 (150.35 to 155.23) | 39.47 (38.84 to 40.10) |  | 153.62 (149.84 to 157.39) | 40.90 (39.89 to 41.91) |  | 154.43 (148.93 to 159.94) | 42.37 (40.85 to 43.89) |  | 155.23 (147.70 to 162.76) | 43.90 (41.77 to 46.04) |  | 156.01 (146.21 to 165.82) | 45.52 (42.65 to 48.39) |  | 156.80 (144.48 to 169.12) | 47.21 (43.48 to 50.93) |  | 157.60 (142.55 to 172.64) | 48.96 (44.27 to 53.66) |  | 158.40 (140.43 to 176.37) | 50.78 (44.99 to 56.56) |  | 159.19 (138.10 to 180.27) | 52.66 (45.66 to 59.66) |  | 159.97 (135.58 to 184.36) | 54.64 (46.28 to 63.00) |  | 160.77 (132.89 to 188.64) | 56.71 (46.84 to 66.58) |
| Female |  |  |  |  |  |  |  |  |  |  |  |  |  |  |  |  |  |  |  |  |  |  |  |  |  |  |  |  |  |  |  |  |
| Global | 244.31 (240.76 to 247.86) | 1,081.32 (1,065.36 to 1,097.29) |  | 245.08 (239.68 to 250.48) | 1,112.93 (1,088.23 to 1,137.63) |  | 245.83 (238.05 to 253.62) | 1,144.96 (1,108.55 to 1,181.37) |  | 246.58 (235.99 to 257.16) | 1,178.01 (1,127.33 to 1,228.68) |  | 247.34 (233.61 to 261.07) | 1,212.45 (1,145.06 to 1,279.84) |  | 248.12 (230.92 to 265.31) | 1,247.93 (1,161.37 to 1,334.49) |  | 248.87 (227.93 to 269.82) | 1,283.87 (1,175.76 to 1,391.98) |  | 249.60 (224.65 to 274.55) | 1,319.92 (1,187.93 to 1,451.91) |  | 250.31 (221.11 to 279.51) | 1,356.63 (1,198.33 to 1,514.93) |  | 251.03 (217.34 to 284.73) | 1,394.49 (1,207.29 to 1,581.69) |  | 251.78 (213.35 to 290.21) | 1,433.09 (1,214.33 to 1,651.84) |
| High SDI | 290.16 (285.30 to 295.03) | 283.70 (278.80 to 288.60) |  | 292.23 (284.77 to 299.70) | 290.83 (283.29 to 298.37) |  | 294.33 (283.46 to 305.20) | 298.06 (286.97 to 309.15) |  | 296.44 (281.54 to 311.34) | 305.41 (289.99 to 320.82) |  | 298.58 (279.12 to 318.04) | 312.90 (292.45 to 333.35) |  | 300.75 (276.23 to 325.27) | 320.50 (294.32 to 346.68) |  | 302.97 (272.93 to 333.01) | 328.15 (295.57 to 360.73) |  | 305.23 (269.24 to 341.23) | 335.74 (296.11 to 375.37) |  | 307.51 (265.15 to 349.88) | 343.30 (295.97 to 390.63) |  | 309.82 (260.67 to 358.97) | 350.88 (295.18 to 406.58) |  | 312.17 (255.81 to 368.52) | 358.43 (293.69 to 423.16) |
| High-middle SDI | 220.21 (215.52 to 224.90) | 132.54 (129.62 to 135.46) |  | 220.61 (213.96 to 227.27) | 135.09 (130.94 to 139.24) |  | 220.95 (211.72 to 230.19) | 137.64 (131.82 to 143.45) |  | 221.29 (209.01 to 233.58) | 140.27 (132.44 to 148.11) |  | 221.66 (205.93 to 237.39) | 143.04 (132.85 to 153.23) |  | 222.03 (202.51 to 241.55) | 145.88 (133.02 to 158.74) |  | 222.30 (198.7 to 245.91) | 148.70 (132.88 to 164.51) |  | 222.51 (194.56 to 250.46) | 151.45 (132.40 to 170.51) |  | 222.72 (190.16 to 255.27) | 154.23 (131.67 to 176.80) |  | 222.94 (185.54 to 260.35) | 157.08 (130.71 to 183.46) |  | 223.17 (180.67 to 265.67) | 159.95 (129.48 to 190.43) |
| Middle  SDI | 251.36 (247.47 to 255.25) | 439.61 (432.66 to 446.55) |  | 252.56 (246.73 to 258.39) | 455.40 (444.77 to 466.02) |  | 253.70 (245.34 to 262.06) | 471.23 (455.61 to 486.85) |  | 254.82 (243.49 to 266.16) | 487.63 (465.87 to 509.40) |  | 256.01 (241.30 to 270.72) | 504.76 (475.70 to 533.81) |  | 257.25 (238.80 to 275.69) | 522.31 (484.81 to 559.80) |  | 258.41 (235.92 to 280.91) | 539.83 (492.80 to 586.85) |  | 259.51 (232.68 to 286.34) | 557.22 (499.60 to 614.84) |  | 260.60 (229.16 to 292.03) | 574.89 (505.54 to 644.23) |  | 261.73 (225.42 to 298.05) | 593.06 (510.78 to 675.33) |  | 262.93 (221.44 to 304.42) | 611.43 (514.97 to 707.89) |
| Low- middle  SDI | 199.77 (195.69 to 203.85) | 185.15 (181.38 to 188.92) |  | 199.86 (193.92 to 205.81) | 190.19 (184.54 to 195.84) |  | 199.92 (191.53 to 208.32) | 195.34 (187.15 to 203.53) |  | 199.95 (188.69 to 211.21) | 200.61 (189.32 to 211.91) |  | 199.95 (185.47 to 214.42) | 206.05 (191.14 to 220.97) |  | 199.93 (181.94 to 217.92) | 211.65 (192.60 to 230.69) |  | 199.90 (178.13 to 221.66) | 217.34 (193.68 to 241.01) |  | 199.83 (174.05 to 225.62) | 223.11 (194.32 to 251.90) |  | 199.74 (169.72 to 229.76) | 228.99 (194.57 to 263.41) |  | 199.63 (165.16 to 234.09) | 235.05 (194.47 to 275.63) |  | 199.52 (160.40 to 238.63) | 241.27 (193.97 to 288.57) |
| Low SDI | 169.94 (167.34 to 172.54) | 45.61 (44.90 to 46.31) |  | 171.14 (166.90 to 175.39) | 47.48 (46.29 to 48.66) |  | 172.33 (165.98 to 178.69) | 49.40 (47.57 to 51.23) |  | 173.50 (164.68 to 182.32) | 51.41 (48.79 to 54.04) |  | 174.64 (163.03 to 186.24) | 53.53 (49.96 to 57.10) |  | 175.77 (161.09 to 190.44) | 55.75 (51.08 to 60.42) |  | 176.90 (158.89 to 194.92) | 58.06 (52.13 to 63.99) |  | 178.04 (156.43 to 199.65) | 60.45 (53.10 to 67.81) |  | 179.15 (153.70 to 204.59) | 62.94 (53.98 to 71.90) |  | 180.24 (150.72 to 209.76) | 65.55 (54.79 to 76.31) |  | 181.33 (147.50 to 215.15) | 68.28 (55.52 to 81.05) |

BAPC, Bayesian age-period-cohort; CKD, chronic kidney disease; CI, confidence interval; SDI, social-demographic index.

**Table S6. BAPC prediction of death of CKD by sex and SDI** in 2020-2030.

|  | 2020 | |  | 2021 | |  | 2022 | |  | 2023 | |  | 2024 | |  | 2025 | |  | 2026 | |  | 2027 | |  | 2028 | |  | 2029 | |  | 2030 | |
| --- | --- | --- | --- | --- | --- | --- | --- | --- | --- | --- | --- | --- | --- | --- | --- | --- | --- | --- | --- | --- | --- | --- | --- | --- | --- | --- | --- | --- | --- | --- | --- | --- |
|  | Rate per 100,000 (95% CI) | Cases No. ×10^4^ (95% CI) |  | Rate per 100,000 (95% CI) | Cases No. ×10^4^ (95% CI) |  | Rate per 100,000 (95% CI) | Cases No. ×10^4^ (95% CI) |  | Rate per 100,000 (95% CI) | Cases No. ×10^4^ (95% CI) |  | Rate per 100,000 (95% CI) | Cases No. ×10^4^ (95% CI) |  | Rate per 100,000 (95% CI) | Cases No. ×10^4^ (95% CI) |  | Rate per 100,000 (95% CI) | Cases No. ×10^4^ (95% CI) |  | Rate per 100,000 (95% CI) | Cases No. ×10^4^ (95% CI) |  | Rate per 100,000 (95% CI) | Cases No. ×10^4^ (95% CI) |  | Rate per 100,000 (95% CI) | Cases No. ×10^4^ (95% CI) |  | Rate per 100,000 (95% CI) | Cases No. ×10^4^ (95% CI) |
| Both sexes |  |  |  |  |  |  |  |  |  |  |  |  |  |  |  |  |  |  |  |  |  |  |  |  |  |  |  |  |  |  |  |  |
| Global | 18.45 (1.23 to 543.11) | 148.38 (145.23 to 151.53) |  | 18.41 (0.73 to 682.90) | 152.50 (147.78 to 157.23) |  | 18.35 (0.19 to 1182.74) | 156.30 (149.50 to 163.09) |  | 18.26 (0.00 to 1960.40) | 160.26 (150.96 to 169.55) |  | 18.13 (0.00 to 2770.22) | 164.66 (152.44 to 176.88) |  | 17.98 (0.00 to 3469.09) | 169.23 (153.66 to 184.80) |  | 17.84 (0.00 to 4026.68) | 173.76 (154.45 to 193.06) |  | 17.71 (0.00 to 4456.46) | 178.10 (154.69 to 201.51) |  | 17.60 (0.00 to 4781.53) | 182.58 (154.67 to 210.49) |  | 17.50 (0.00 to 5024.30) | 187.42 (154.54 to 220.30) |  | 17.41 (0.00 to 5203.99) | 192.42 (154.11 to 230.73) |
| High SDI | 12.56 (7.31 to 49.97) | 26.90 (26.20 to 27.60) |  | 12.50 (5.92 to 69.45) | 27.52 (26.42 to 28.62) |  | 12.44 (4.05 to 125.62) | 28.01 (26.40 to 29.62) |  | 12.37 (3.06 to 203.76) | 28.53 (26.31 to 30.76) |  | 12.32 (2.31 to 282.83) | 29.16 (26.22 to 32.10) |  | 12.26 (2.03 to 352.01) | 29.81 (26.05 to 33.56) |  | 12.20 (1.86 to 408.49) | 30.43 (25.78 to 35.08) |  | 12.15 (1.74 to 452.74) | 31.00 (25.37 to 36.63) |  | 12.10 (1.63 to 486.30) | 31.61 (24.91 to 38.30) |  | 12.07 (1.55 to 511.07) | 32.30 (24.44 to 40.17) |  | 12.05 (1.48 to 528.91) | 33.03 (23.88 to 42.18) |
| High-middle SDI | 12.06 (6.37 to 56.39) | 13.83 (13.33 to 14.32) |  | 12.08 (5.77 to 70.65) | 14.22 (13.53 to 14.90) |  | 12.10 (4.38 to 119.39) | 14.55 (13.61 to 15.49) |  | 12.11 (3.15 to 202.91) | 14.90 (13.65 to 16.16) |  | 12.12 (2.36 to 300.70) | 15.31 (13.69 to 16.94) |  | 12.12 (1.87 to 394.31) | 15.74 (13.68 to 17.79) |  | 12.11 (1.56 to 474.63) | 16.14 (13.61 to 18.67) |  | 12.10 (1.42 to 539.38) | 16.51 (13.46 to 19.56) |  | 12.09 (1.32 to 589.57) | 16.90 (13.28 to 20.52) |  | 12.09 (1.25 to 627.62) | 17.33 (13.09 to 21.58) |  | 12.08 (1.20 to 656.06) | 17.78 (12.84 to 22.71) |
| Middle  SDI | 23.73 (15.31 to 61.28) | 73.54 (71.37 to 75.70) |  | 23.80 (14.80 to 66.47) | 76.26 (73.23 to 79.29) |  | 23.86 (12.87 to 88.57) | 78.89 (74.68 to 83.09) |  | 23.91 (10.03 to 136.48) | 81.64 (75.97 to 87.30) |  | 23.96 (7.76 to 202.72) | 84.63 (77.22 to 92.04) |  | 24.00 (6.18 to 272.43) | 87.74 (78.32 to 97.17) |  | 24.03 (5.18 to 335.49) | 90.86 (79.15 to 102.58) |  | 24.04 (4.45 to 388.02) | 93.93 (79.67 to 108.18) |  | 24.06 (4.09 to 429.80) | 97.10 (80.01 to 114.19) |  | 24.07 (3.86 to 462.16) | 100.49 (80.25 to 120.74) |  | 24.09 (3.68 to 486.84) | 104.00 (80.27 to 127.72) |
| Low- middle  SDI | 23.21 (13.74 to 60.39) | 36.95 (35.87 to 38.04) |  | 23.24 (12.13 to 72.17) | 38.09 (36.44 to 39.74) |  | 23.26 (8.16 to 119.63) | 39.21 (36.81 to 41.62) |  | 23.27 (5.09 to 199.89) | 40.38 (37.05 to 43.71) |  | 23.28 (3.28 to 285.55) | 41.64 (37.22 to 46.05) |  | 23.31 (2.36 to 358.97) | 42.95 (37.28 to 48.61) |  | 23.34 (1.91 to 416.32) | 44.28 (37.21 to 51.35) |  | 23.37 (1.68 to 459.45) | 45.61 (36.98 to 54.24) |  | 23.41 (1.51 to 491.31) | 45.61 (36.98 to 54.24) |  | 23.45 (1.38 to 514.45) | 48.43 (36.16 to 60.70) |  | 23.51 (1.29 to 530.93) | 49.94 (35.57 to 64.31) |
| Low SDI | 25.26 (14.55 to 69.10) | 10.34 (10.13 to 10.55) |  | 25.13 (12.63 to 85.01) | 10.63 (10.32 to 10.95) |  | 25.00 (9.04 to 138.06) | 10.93 (10.47 to 11.38) |  | 24.86 (6.39 to 210.85) | 11.23 (10.61 to 11.86) |  | 24.73 (4.94 to 279.24) | 11.57 (10.74 to 12.39) |  | 24.60 (3.91 to 334.03) | 11.91 (10.86 to 12.97) |  | 24.47 (3.13 to 375.56) | 12.27 (10.96 to 13.58) |  | 24.35 (2.76 to 406.47) | 12.63 (11.04 to 14.23) |  | 24.23 (2.52 to 429.27) | 13.01 (11.10 to 14.93) |  | 24.12 (2.33 to 445.91) | 13.42 (11.15 to 15.69) |  | 24.01 (2.17 to 457.87) | 13.84 (11.19 to 16.50) |
| Male |  |  |  |  |  |  |  |  |  |  |  |  |  |  |  |  |  |  |  |  |  |  |  |  |  |  |  |  |  |  |  |  |
| Global | 21.68 (21.28 to 22.07) | 77.27 (75.77 to 78.76) |  | 21.60 (21.00 to 22.20) | 79.31 (77.03 to 81.59) |  | 21.52 (20.66 to 22.38) | 81.19 (77.89 to 84.48) |  | 21.43 (20.28 to 22.59) | 83.16 (78.63 to 87.68) |  | 21.35 (19.87 to 22.84) | 85.35 (79.39 to 91.31) |  | 21.27 (19.43 to 23.11) | 87.64 (80.04 to 95.24) |  | 21.19 (18.97 to 23.41) | 89.89 (80.46 to 99.32) |  | 21.10 (18.48 to 23.72) | 92.04 (80.61 to 103.47) |  | 21.01 (17.97 to 24.04) | 94.25 (80.63 to 107.88) |  | 20.91 (17.45 to 24.38) | 96.66 (80.61 to 112.70) |  | 20.82 (16.90 to 24.74) | 99.14 (80.46 to 117.83) |
| High SDI | 15.21 (14.85 to 15.57) | 12.99 (12.66 to 13.31) |  | 15.11 (14.55 to 15.66) | 13.31 (12.81 to 13.81) |  | 15.00 (14.20 to 15.80) | 13.59 (12.86 to 14.32) |  | 14.90 (13.83 to 15.98) | 13.89 (12.88 to 14.89) |  | 14.81 (13.43 to 16.20) | 14.24 (12.91 to 15.57) |  | 14.73 (13.01 to 16.45) | 14.61 (12.90 to 16.31) |  | 14.64 (12.57 to 16.71) | 14.96 (12.84 to 17.08) |  | 14.54 (12.10 to 16.98) | 15.28 (12.71 to 17.84) |  | 14.45 (11.62 to 17.29) | 15.62 (12.56 to 18.68) |  | 14.38 (11.14 to 17.62) | 16.01 (12.40 to 19.62) |  | 14.30 (10.64 to 17.97) | 16.41 (12.21 to 20.62) |
| High-middle SDI | 14.94 (14.54 to 15.35) | 6.44  (6.26 to 6.62) |  | 14.92 (14.31 to 15.52) | 6.60  (6.33 to 6.88) |  | 14.88 (14.02 to 15.74) | 6.74  (6.35 to 7.13) |  | 14.85 (13.69 to 16.00) | 6.89  (6.35 to 7.43) |  | 14.82 (13.33 to 16.30) | 7.06  (6.35 to 7.77) |  | 14.78 (12.94 to 16.62) | 7.24  (6.34 to 8.15) |  | 14.74 (12.51 to 16.96) | 7.41  (6.29 to 8.54) |  | 14.69 (12.06 to 17.32) | 7.57  (6.21 to 8.93) |  | 14.64 (11.58 to 17.69) | 7.74  (6.12 to 9.35) |  | 14.58 (11.09 to 18.08) | 7.92  (6.02 to 9.82) |  | 14.52 (10.57 to 18.48) | 8.12  (5.91 to 10.33) |
| Middle  SDI | 26.98 (26.31 to 27.64) | 38.40 (37.45 to 39.36) |  | 27.02 (26.11 to 27.92) | 39.71 (38.37 to 41.04) |  | 27.05 (25.82 to 28.27) | 40.95 (39.09 to 42.80) |  | 27.07 (25.47 to 28.67) | 42.24 (39.74 to 44.74) |  | 27.09 (25.07 to 29.11) | 43.66 (40.40 to 46.92) |  | 27.11 (24.63 to 29.60) | 45.13 (40.99 to 49.27) |  | 27.12 (24.14 to 30.10) | 46.60 (41.47 to 51.73) |  | 27.11 (23.60 to 30.63) | 48.02 (41.79 to 54.24) |  | 27.10 (23.02 to 31.17) | 49.48 (42.05 to 56.92) |  | 27.07 (22.42 to 31.73) | 51.06 (42.28 to 59.84) |  | 27.05 (21.78 to 32.31) | 52.69 (42.42 to 62.95) |
| Low- middle  SDI | 27.11 (26.32 to 27.91) | 20.61  (20.00 to 21.22) |  | 27.13 (25.95 to 28.31) | 21.24 (20.31 to 22.17) |  | 27.14 (25.46 to 28.82) | 21.86 (20.50 to 23.22) |  | 27.15 (24.89 to 29.42) | 22.50 (20.63 to 24.38) |  | 27.16 (24.24 to 30.08) | 23.20 (20.70 to 25.69) |  | 27.18 (23.54 to 30.81) | 23.92 (20.72 to 27.12) |  | 27.20 (22.79 to 31.60) | 24.66 (20.67 to 28.66) |  | 27.22 (21.99 to 32.44) | 25.40 (20.52 to 30.28) |  | 27.23 (21.13 to 33.33) | 26.16 (20.30 to 32.02) |  | 27.24 (20.23 to 34.26) | 26.97 (20.03 to 33.91) |  | 27.26 (19.29 to 35.24) | 27.81 (19.67 to 35.94) |
| Low SDI | 29.08 (28.54 to 29.63) | 5.74  (5.62 to 5.85) |  | 28.87 (28.06 to 29.68) | 5.89  (5.72 to 6.05) |  | 28.67 (27.53 to 29.80) | 6.03  (5.79 to 6.27) |  | 28.46 (26.95 to 29.97) | 6.18  (5.85 to 6.51) |  | 28.26 (26.34 to 30.18) | 6.35  (5.92 to 6.78) |  | 28.06 (25.69 to 30.43) | 6.52  (5.97 to 7.08) |  | 27.87 (25.03 to 30.71) | 6.70  (6.02 to 7.39) |  | 27.68 (24.34 to 31.02) | 6.88  (6.05 to 7.71) |  | 27.50 (23.64 to 31.36) | 7.07  (6.08 to 8.07) |  | 27.32 (22.92 to 31.72) | 7.28  (6.10 to 8.45) |  | 27.14 (22.18 to 32.10) | 7.49  (6.12 to 8.86) |
| Female |  |  |  |  |  |  |  |  |  |  |  |  |  |  |  |  |  |  |  |  |  |  |  |  |  |  |  |  |  |  |  |  |
| Global | 15.93 (15.58 to 16.28) | 71.11 (69.46 to 72.77) |  | 15.92 (15.40 to 16.44) | 73.19 (70.74 to 75.65) |  | 15.90 (15.17 to 16.63) | 75.11 (71.61 to 78.61) |  | 15.88 (14.90 to 16.85) | 77.10 (72.33 to 81.87) |  | 15.85 (14.60 to 17.09) | 79.31 (73.05 to 85.57) |  | 15.82 (14.28 to 17.36) | 81.59 (73.63 to 89.56) |  | 15.78 (13.93 to 17.64) | 83.87 (73.99 to 93.75) |  | 15.74 (13.55 to 17.93) | 86.06 (74.08 to 98.04) |  | 15.69 (13.16 to 18.23) | 88.33 (74.04 to 102.61) |  | 15.64 (12.74 to 18.54) | 90.77 (73.93 to 107.60) |  | 15.58 (12.30 to 18.85) | 93.28 (73.66 to 112.90) |
| High SDI | 10.63 (10.36 to 10.91) | 13.91 (13.53 to 14.29) |  | 10.59 (10.15 to 11.03) | 14.20 (13.61 to 14.8) |  | 10.54 (9.90 to 11.18) | 14.42 (13.54 to 15.30) |  | 10.49 (9.63 to 11.36) | 14.64 (13.43 to 15.86) |  | 10.45 (9.33 to 11.57) | 14.92 (13.31 to 16.52) |  | 10.41 (9.01 to 11.80) | 15.20 (13.15 to 17.25) |  | 10.36 (8.67 to 12.06) | 15.47 (12.94 to 18.01) |  | 10.32 (8.31 to 12.32) | 15.72 (12.67 to 18.78) |  | 10.28 (7.95 to 12.61) | 15.99 (12.36 to 19.62) |  | 10.24 (7.57 to 12.92) | 16.30 (12.04 to 20.56) |  | 10.21 (7.17 to 13.24) | 16.62 (11.68 to 21.56) |
| High-middle SDI | 10.32 (9.91 to 10.73) | 7.39  (7.08 to 7.70) |  | 10.36 (9.82 to 10.90) | 7.61  (7.20 to 8.03) |  | 10.39 (9.68 to 11.11) | 7.81  (7.26 to 8.36) |  | 10.43 (9.51 to 11.35) | 8.02  (7.30 to 8.73) |  | 10.46 (9.30 to 11.61) | 8.25  (7.33 to 9.17) |  | 10.48 (9.07 to 11.89) | 8.49  (7.34 to 9.65) |  | 10.49 (8.80 to 12.18) | 8.73  (7.32 to 10.14) |  | 10.50 (8.52 to 12.49) | 8.94  (7.25 to 10.64) |  | 10.51 (8.21 to 12.80) | 9.17  (7.16 to 11.17) |  | 10.50 (7.89 to 13.12) | 9.41  (7.06 to 11.76) |  | 10.49 (7.54 to 13.44) | 9.66  (6.94 to 12.38) |
| Middle  SDI | 21.04 (20.32 to 21.76) | 35.13 (33.92 to 36.34) |  | 21.13 (20.15 to 22.10) | 36.55 (34.86 to 38.24) |  | 21.20 (19.89 to 22.51) | 37.94 (35.60 to 40.29) |  | 21.27 (19.56 to 22.97) | 39.40 (36.23 to 42.56) |  | 21.33 (19.17 to 23.48) | 40.97 (36.83 to 45.12) |  | 21.37 (18.73 to 24.02) | 42.61 (37.33 to 47.89) |  | 21.41 (18.23 to 24.59) | 44.27 (37.69 to 50.85) |  | 21.44 (17.69 to 25.18) | 45.91 (37.88 to 53.94) |  | 21.45 (17.11 to 25.79) | 47.61 (37.97 to 57.26) |  | 21.46 (16.48 to 26.43) | 49.43 (37.97 to 60.89) |  | 21.45 (15.82 to 27.07) | 51.31 (37.85 to 64.77) |
| Low- middle  SDI | 19.74 (19.17 to 20.31) | 16.34 (15.87 to 16.82) |  | 19.76 (18.92 to 20.61) | 16.85 (16.13 to 17.57) |  | 19.78 (18.59 to 20.98) | 17.35 (16.31 to 18.40) |  | 19.80 (18.20 to 21.40) | 17.88 (16.43 to 19.32) |  | 19.81 (17.75 to 21.87) | 18.44 (16.52 to 20.36) |  | 19.82 (17.25 to 22.38) | 19.02 (16.56 to 21.48) |  | 19.83 (16.72 to 22.93) | 19.62 (16.54 to 22.69) |  | 19.83 (16.15 to 23.51) | 20.21 (16.46 to 23.96) |  | 19.83 (15.54 to 24.11) | 20.82 (16.32 to 25.32) |  | 19.82 (14.90 to 24.74) | 21.46 (16.13 to 26.79) |  | 19.81 (14.23 to 25.40) | 22.13 (15.89 to 28.37) |
| Low SDI | 21.74 (21.34 to 22.15) | 4.60  (4.51 to 4.69) |  | 21.69 (21.06 to 22.33) | 4.75  (4.60 to 4.89) |  | 21.64 (20.72 to 22.56) | 4.89  (4.68 to 5.11) |  | 21.59 (20.34 to 22.83) | 5.05  (4.76 to 5.34) |  | 21.53 (19.93 to 23.13) | 5.22  (4.83 to 5.61) |  | 21.47 (19.48 to 23.45) | 5.39  (4.89 to 5.89) |  | 21.40 (19.00 to 23.8) | 5.57  (4.94 to 6.20) |  | 21.34 (18.50 to 24.17) | 5.75  (4.99 to 6.52) |  | 21.27 (17.97 to 24.56) | 5.94  (5.02 to 6.86) |  | 21.20 (17.42 to 24.97) | 6.14  (5.05 to 7.24) |  | 21.12 (16.85 to 25.39) | 6.35  (5.07 to 7.64) |

BAPC, Bayesian age-period-cohort; CKD, chronic kidney disease; CI, confidence interval; SDI, social-demographic index.

**Table S7. Previous studies using the GBD database to analyze the epidemiology of CKD.**

| Authors | Date of publication | Causes of CKD | Region | Methods | Conclusion | PMID |
| --- | --- | --- | --- | --- | --- | --- |
| Qing et al. | 2024 | All | Global | Joinpoint regression model | There is great heterogeneity in the prevalence and DALYs of CKD of different causes worldwide, and the distribution of CKD varies significantly | 38512378 |
| Ying et al. | 2024 | All | Global | Descriptive analysis | From 1990 to 2019, the global incidence of CKD more than doubled, and DALYs nearly doubled, exceeding 40 million per year. CKD caused by diabetes and hypertension accounted for nearly two-thirds of DALYs of known causes in 2019 | 37717572 |
| Song et al. | 2024 | Lead exposure | Global | Joinpoint regression model | A large proportion of the burden of CKD was attributable to lead exposure, with marked regional disparities | 37907111 |
| Rashidi et al. | 2024 | All | Global | Descriptive analysis | Significant disparities in CKD care quality remain, with Europe having the highest quality of care index scores and Africa having the lowest quality of care index scores | 37587021 |
| Ren et al. | 2024 | Hypertension | Global | APC model; Nordpred APC analysis | From 1990 to 2019, the ASIR of hypertension-related CKD showed an upward trend, and it is predicted that it will continue to rise in the next 25 years | 37542001 |
| Wei et al. | 2024 | High fasting plasma glucose | Global | Descriptive analysis | The burden of CKD attributable to high fasting plasma glucose was expected to increase as populations grow and dietary patterns change. From 1990 to 2019, the burden was greater in males and in developing regions | 38601204 |
| Zhao et al. | 2024 | All | Global | Descriptive analysis | The global burden of CKD among children and adolescents is increasing, especially in regions and countries with lower SDI | 38130213 |
| Deng et al. | 2024 | All | Global | Descriptive analysis | CKD is a growing global health problem in adolescents and young adults, especially in countries with a middle SDI | 39034860 |
| Liu et al. | 2024 | Type 2 diabetes | Global | APC model | From 1990 to 2019, the global burden of CKD in type 2 diabetes showed an overall upward trend, with the highest burden in medium SDI regions and the lowest in low SDI regions | 37936340 |
| Dávila-Cervantes et al. | 2024 | All | Mexico | Descriptive analysis | The burden of young-onset chronic CKD has increased unprecedentedly in the Mexican population over the past 30 years | 38387518 |
| Feng et al. | 2023 | All | Global | Joinpoint regression model | CKD-related deaths occur in the elderly, and the burden of CKD-related deaths in men is higher than that in women. The increased burden of heat-related deaths in low SDI areas should arouse social concern | 36931681 |
| Hu et al. | 2023 | Glomerulonephritis | Global | Decomposition analysis; frontier Analysis | The burden of CKD caused by glomerulonephritis is increasing worldwide, especially in the low SDI regions | 36719159 |
| Xie et al. | 2023 | Type 2 diabetes | Global | APC model; decomposition analysis | CKD due to Type 2 Diabetes has become a growing public health problem worldwide, especially for adults under 60 years old, with a higher disease burden in men than in women Population growth and aging are important drivers of the increase in DALYs burden of CKD, among which high BMI and high systolic blood pressure are considered to be the main modifiable risk factors | 37981642 |
| Liu et al. | 2023 | High sodium intake | Global | Descriptive analysis | There are significant sexual and geographic variations in the burden of CKD attributable to high sodium intake and its temporal trends. Globally, the high sodium intake-caused CKD burden continues to elevate, posing a major challenge to public health | 36937353 |
| Liu et al. | 2023 | Hypertension | Global | Joinpoint regression model; spatial autocorrelation analysis | The global burden of hypertension-related CKD is increasing from 1990 to 2019, and the AAPC of ASDR and ASIR have significant positive spatial autocorrelation | 38031057 |
| Jiang et al. | 2023 | Glomerulonephritis | Global | Descriptive analysis | The burden of CKD due to glomerulonephritis is disproportionately higher in developing countries and less developed economies, with gender differences | 36564906 |
| Gui et al. | 2023 | Lead exposure | Global | Joinpoint regression model; APC model | From 1990 to 2019, the global burden of CKD due to lead exposure increased, especially in low SDI and medium-low SDI areas and in the elderly | 37669716 |
| GBD 2019 Pakistan Collaborators | 2023 | All | Pakistan | Decomposition analysis | Pakistan has made progress in reducing its disease burden since 1990, but geographic, age, and gender disparities persist | 36669807 |
| Hockham et al. | 2022 | All | Global | Descriptive analysis | From 1990 to 2019, in most regions, the CKD ASMR among female continued to be lower than that among male, and the percentage change in ASMR was lower among female than among male | 36159166 |
| Li et al. | 2022 | All | China | Joinpoint regression model; APC model; ARIMA model | The prevalence and mortality rates of CKD in China increased significantly from 1990 to 2019. By 2029, the prevalence and mortality rates of CKD are expected to rise to 11.7% and 17.1 per 100,000 people, respectively | 36755850 |
| Aashima et al. | 2022 | All | Asia | Descriptive analysis | CKD is common in Asia and is a heavy burden in resource-poor countries | 35506615 |
| Ke et al. | 2022 | All | Low-and middle-income regions | Descriptive analysis | The burden of CKD remains high across all income regions, especially in low- and middle-income countries | 34986789 |
| Jian et al. | 2022 | All | China | ARIMA model | The number of CKD patients in China and the economic burden of CKD will continue to rise. From 2020 to 2025, the total economic burden of CKD will increase by an average of $3.1 billion per year per year | 36585665 |
| Wen et al. | 2022 | All | China, Japan, the United Kingdom, and the United States | Decomposition analysis | From 1990 to 2019, CKD DALY in China, Japan, the UK, and the US all showed an upward trend, with population growth and population aging contributing significantly | 36159316 |
| Tabatabaei-Malazy et al. | 2022 | All | North Africa and Middle East region | Descriptive analysis | The burden and prevalence of CKD in the North Africa and Middle East region have increased alarmingly, especially in males and those aged between 70 and 74 years. Furthermore, the three major risk factors contributing to the burden of CKD are renal dysfunction, hypertension, and high BMI | 36304241 |
| Pan et al. | 2022 | Diabetes | China | Descriptive analysis | The burden of diabetes-related CKD in China has increased, with gender and age differences | 35784566 |
| Deng et al. | 2021 | Diabetes | Global | Descriptive analysis | Diabetes was the leading cause of new CKD cases and patients in all regions, and the growth of diabetes-related CKD burden varied in different regions and countries | 34276558 |
| GBD Chronic Kidney Disease Collaboration | 2020 | All | Global | Descriptive analysis | CKD is a major global health concern, contributing to morbidity, mortality, and increased risk of cardiovascular diseases | 32061315 |
| Xie et al. | 2018 | All | Global | Decomposition analysis; Frontier Analysis | The global burden of CKD is large, rising, and unevenly distributed; it is driven primarily by population expansion and large increases in diabetes in some regions. Opportunities to reduce the burden of CKD exist at all stages of development | 30078514 |

GBD, Global Burden of Disease; CKD, chronic kidney disease; DALYs; disability-adjusted life years; APC, Age-Period-Cohort; ASIR, age-standardized incidence rate; ARIMA, autoregressive integrated moving average; SDI, social-demographic index; BMI, body mass index; ASMR, age-standardized mortality rate; AAPC, Average Annual Percent Change; ASDR, age-standardized disability-adjusted life year rate.

**
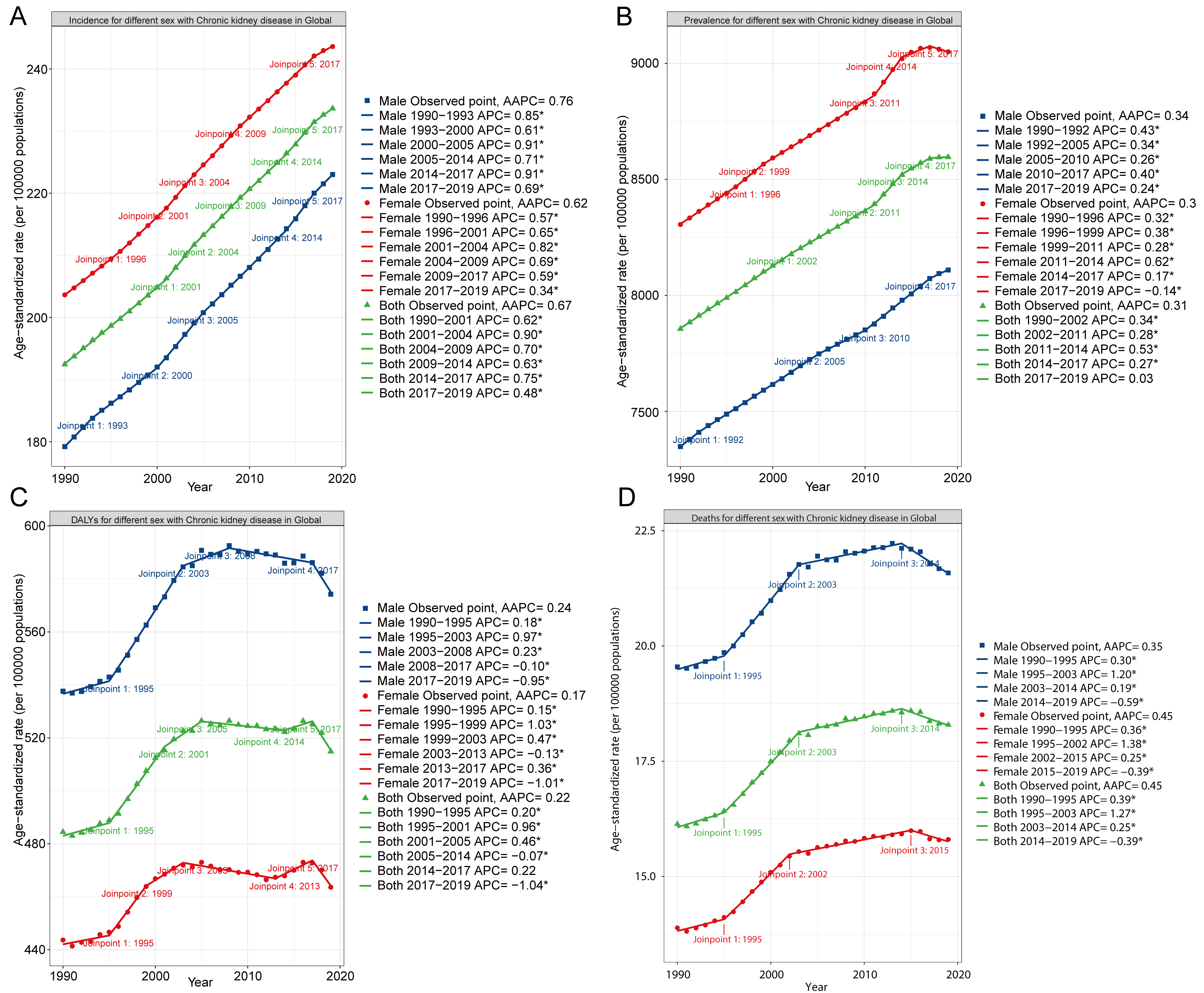
**

**Figure S1. Joinpoint analysis of (A) ASIR, (B) ASPR, (C) ASMR, and (D) ASDR of CKD in the globe from 1990 to 2019.** ASIR, age-standardized incidence rate; ASPR, age-standardized prevalence rate; ASMR, age-standardized mortality rate; ASDR, age-standardized disability-adjusted life year rate; CKD, chronic kidney disease.

**
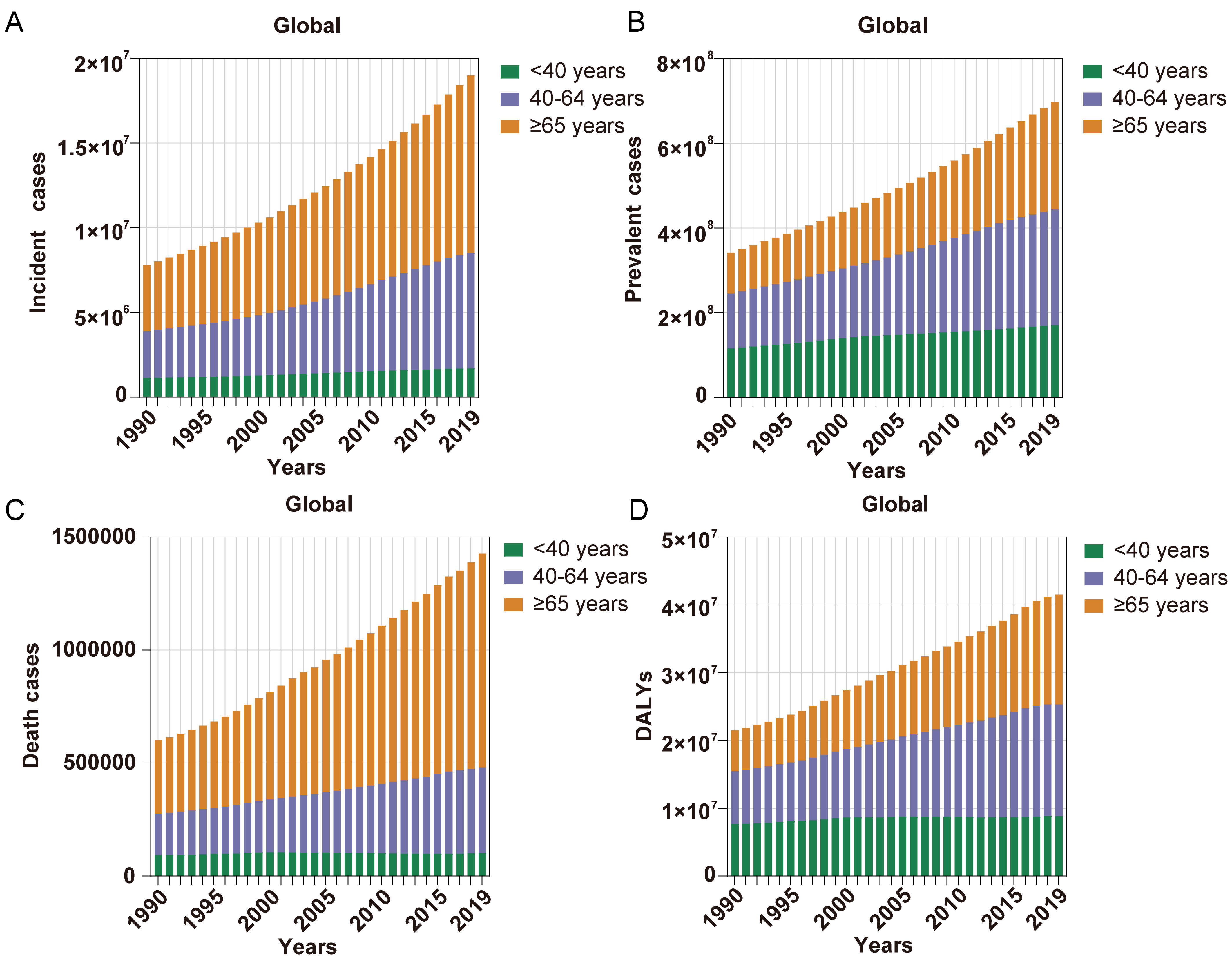
**

**Figure S2. The incident cases (A), prevalent cases (B), deaths (C) and DALYs (D) of CKD in the globe by age, 1990-2019.** DALYs, disability-adjusted life years; CKD, chronic kidney disease.


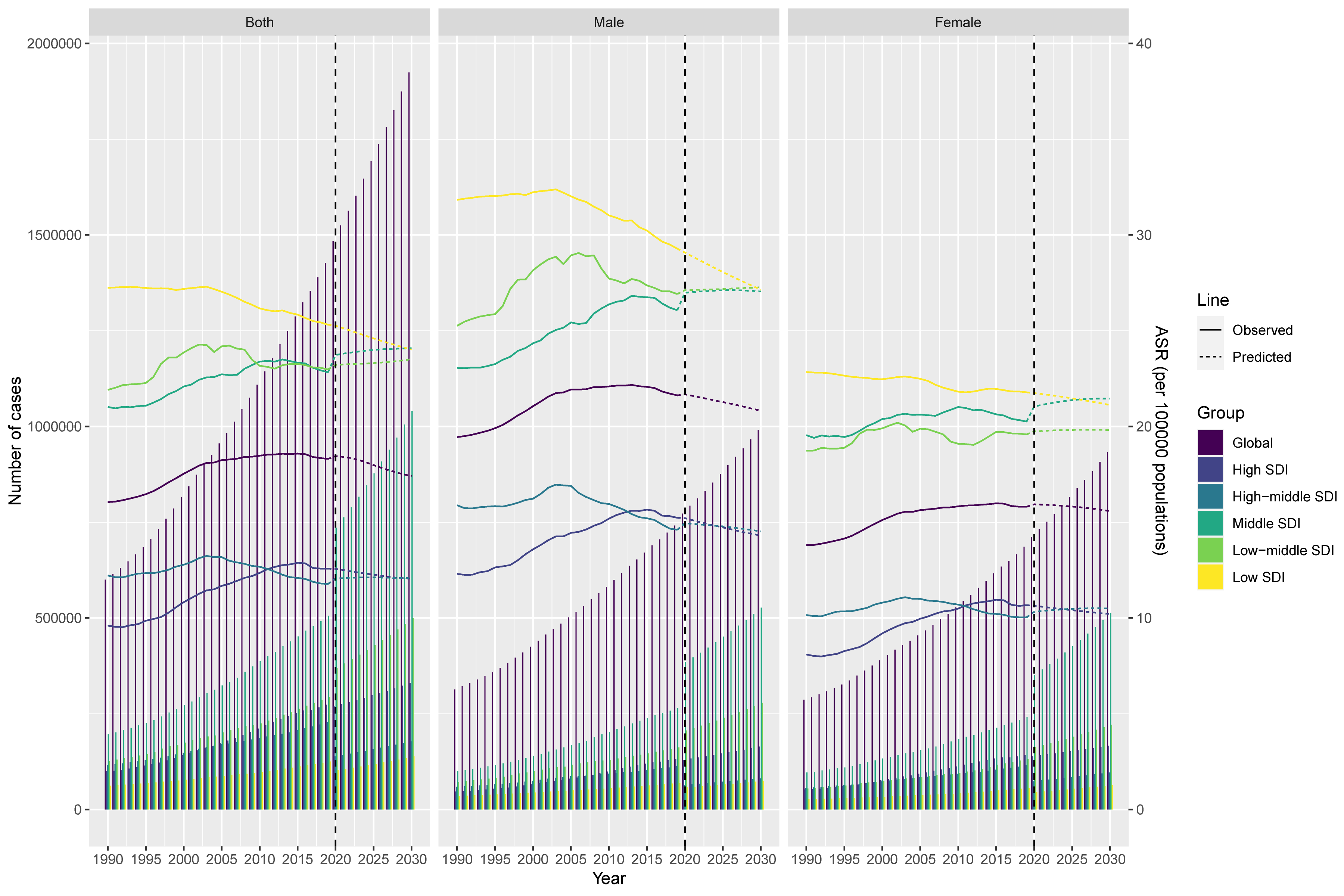
 **Figure S3. The trends of deaths and ASMR in CKD in the globe and five SDI regions by sex, 1990-2030.** ASMR, age-standardized mortality rate; CKD, chronic kidney disease; SDI, sociodemographic index; ASR, age-standardized rate.
